# Comprehensive RNA-Seq Expression Analysis of Sensory Ganglia with a Focus on Ion Channels and GPCRs in Trigeminal Ganglia

**DOI:** 10.1371/journal.pone.0079523

**Published:** 2013-11-08

**Authors:** Stavros Manteniotis, Ramona Lehmann, Caroline Flegel, Felix Vogel, Adrian Hofreuter, Benjamin S. P. Schreiner, Janine Altmüller, Christian Becker, Nicole Schöbel, Hanns Hatt, Günter Gisselmann

**Affiliations:** 1 Department of Cell Physiology, Ruhr-University Bochum, Bochum, Germany; 2 Leibniz Research Center for Working Environment and Human Factors, Dortmund, Germany; 3 Cologne Center for Genomics, University of Cologne, Cologne, Germany; 4 Department of Animal Physiology, Ruhr-University Bochum, Bochum, Germany; Xuzhou Medical college, China

## Abstract

The specific functions of sensory systems depend on the tissue-specific expression of genes that code for molecular sensor proteins that are necessary for stimulus detection and membrane signaling. Using the Next Generation Sequencing technique (RNA-Seq), we analyzed the complete transcriptome of the trigeminal ganglia (TG) and dorsal root ganglia (DRG) of adult mice. Focusing on genes with an expression level higher than 1 FPKM (fragments per kilobase of transcript per million mapped reads), we detected the expression of 12984 genes in the TG and 13195 in the DRG. To analyze the specific gene expression patterns of the peripheral neuronal tissues, we compared their gene expression profiles with that of the liver, brain, olfactory epithelium, and skeletal muscle. The transcriptome data of the TG and DRG were scanned for virtually all known G-protein-coupled receptors (GPCRs) as well as for ion channels. The expression profile was ranked with regard to the level and specificity for the TG. In total, we detected 106 non-olfactory GPCRs and 33 ion channels that had not been previously described as expressed in the TG. To validate the RNA-Seq data, *in situ* hybridization experiments were performed for several of the newly detected transcripts. To identify differences in expression profiles between the sensory ganglia, the RNA-Seq data of the TG and DRG were compared. Among the differentially expressed genes (> 1 FPKM), 65 and 117 were expressed at least 10-fold higher in the TG and DRG, respectively. Our transcriptome analysis allows a comprehensive overview of all ion channels and G protein-coupled receptors that are expressed in trigeminal ganglia and provides additional approaches for the investigation of trigeminal sensing as well as for the physiological and pathophysiological mechanisms of pain.

## Introduction

Sensory neurons that arise from cell bodies of the trigeminal ganglia (TG) and dorsal root ganglia (DRG) are known to detect a large variety of chemical agents and physical stimuli. The DRG are located along the vertebral column. A wide range of specialized neurons detect somatosensory stimuli at the periphery and convey them to the central nervous system. The TG are the cranial analogs of the DRG and are located at the base of the skull (in front of the pons), extending sensory fibers that terminate as free nerve endings in the facial skin and mucosa [1]. By stimulating these neurons, chemical cues can induce a variety of different sensations such as the cooling of menthol, tingling by sanshools, burning and stinging by acids or pungency by capsaicin and mustard oil [2–5]. The trigeminal system and the DRG are known to act as the pain and warning system in mammals. 

Previously, several classes of membrane receptors and ion channels that are critical for trigeminal sensory perception and pathophysiological pain behavior have been described and studied on a molecular level. Much attention has been focused on transient receptor potential (Trp) and potassium channels that act as sensors of temperature, pain, and chemical stimuli [6–8]. Furthermore, nicotinic acetylcholine receptors (nAChRs) that sense nicotine, and voltage-gated sodium channels (VGSCs) important for pain perception and signal transmission, drew considerable attention [9–14]. Today, G protein-coupled receptors and ion channels represent two of the most important targets for pharmacologically active substances [15–17], and the expression pattern of these receptors and ion channels remains to be fully understood. In a recent gene expression study in mice, it has been shown that an alteration of the common gene expression levels for ion channels can be linked to pathophysiological pain diseases [18].

In addition to ion channels, the superfamily of G-protein-coupled receptors (GPCRs) plays a central role in the modulation of pain transmission [19] and in detecting a large range of chemicals [20]. GPCRs are the largest superfamily of cell surface proteins and have seven transmembrane segments as their structural hallmark [21]. These membrane-integral receptor proteins can be activated by either exogenous ligands, such as odorants and taste substances, or by endogenous ligands, such as neurotransmitters, hormones, and inflammatory substances. The receptor family of GPCRs plays a major role in physiological and pathophysiological processes [22,23], and approximately 40-60% of all current drugs target receptors of this class [15,24]. Several classes of GPCRs that are critical for trigeminal pain and histamine-independent pruritus have been identified, including P2Y, opioid receptors, and Mas-related receptors [25–27]. There remain many orphan GPCRs that may play important roles in several physiological functions [28]. 

The trigeminal system is involved in a variety of cranial nerve diseases such as trigeminal neuralgia or neuropathic pain [29–31]. Common causes of neuropathic pain are diabetic neuropathy, nerve compression syndromes, trigeminal neuralgia, stroke, multiple sclerosis, and spinal cord injury [32,33]. Chronic pain remains a major clinical challenge that can significantly diminish the quality of life in affected individuals [34]. 

To fully understand the mechanisms of chemosensation and nociception, it is necessary to analyze the transcriptome of the sensory ganglia and to describe comprehensive gene expression patterns for all ion channels and GPCRs. 

During the last few years, a dynamic development in transcriptome analysis by Next Generation Sequencing (RNA-Seq), in combination with rapidly dropping costs, led to a revolutionary extension of available experimental approaches in transcriptome analysis [35–39]. In contrast to previously used tools, such as microarray analysis, RNA-Seq enables higher resolution measurements of expression [39]. RNA-Seq is a paradigm-shifting technology because of its great sensitivity, highly accurate quantification of expression levels, high dynamic range, and its potential to analyze transcriptomes independently of existing genome annotations. 

However, no attempts have thus far been made to systematically describe the mammalian TG and DRG transcriptome and to characterize their complete ion channel and GPCR expression patterns. 

We used RNA-Seq to analyze the murine TG and DRG transcriptome and to compare the expression profiles of the TG and DRG. Ion channels and GPCRs were ranked according to their expression level and tissue specificity. We primarily detected all important receptors and ion channels with known trigeminal functions that are highly or specifically expressed in the TG. Furthermore, we were able to identify the expression of GPCRs, some of their major signaling compounds, and ion channels whose expression in the TG had not been described before, and we verified their expression by *in situ* hybridization. A differential transcriptome analysis of the TG and DRG identified transcripts that were specific for either of these neuronal tissues. 

## Results and Discussion

### Transcriptome Data

**Figure 1:**
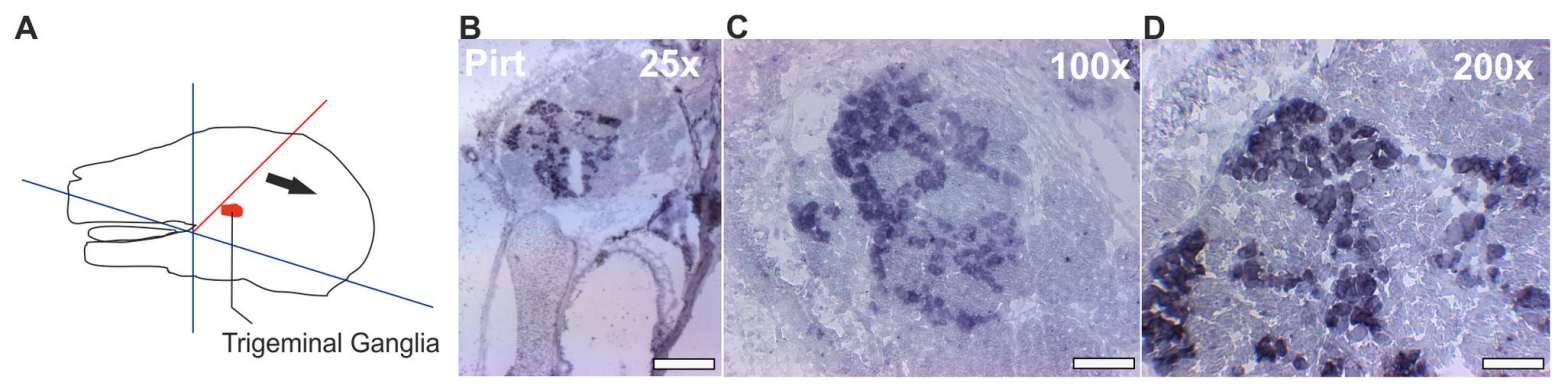
Representative *in* *situ* **hybridization slices from adult mice**. **A** Schematic overview for the preparation of the mouse head slices (14 µm). The nose and the mandible were removed before the slices were prepared (blue line). The black arrow indicates the cutting direction, while the red line indicates the intersecting plane that was used. **B** *In* *situ* hybridization for Pirt mRNA (25-fold). **C** 100-fold. **D** 200-fold magnification of the TG segment. Single cells are strongly stained. Scale bar B: 560 µm, C: 140 µm D: 75 µm.

Using the Illumina Genome Analyzer Gα_IIx_, approximately 36 million and 37 million 36-nucleotide (nt) reads were generated for the TG and DRG by RNA-Seq, respectively. Both tissues contain heterogeneous populations of neurons, such as mechanosensitive, temperature-responding, and nociceptive neurons, as well as glial cells. Each sample was a pool of RNA from 8 male mice (~P28). The sequencing results were analyzed by the TopHat and Cufflinks software. The reads were mapped onto the mouse reference genome (mm9). From the sequenced fragments, 80- 86% could be aligned for both tissues (Table 1). The expression values were calculated for each sample based on the number of fragments per kilobase of exon per million reads mapped (FPKM) [40]. As an approximation, 1 FPKM corresponds to weak expression, 10 FPKM to moderate expression, and 100 FPKM to high expression. As a basis for comparison, we calculated the FPKM values for typical housekeeping genes. For example, the strongly expressed β-actin gene yields an expression value between ~100-1000 FPKM, whereas the weakly to moderately expressed TATA box binding protein (Tbp) is detected at approximately 3-10 FPKM (Figure S1). For an overview of FPKM values for the expression of different genes, we calculated a histogram of the FPKM value distribution for the DRG and TG tissues (Figure S2). Our analysis detected the expression of 16034 genes in the TG and 15946 genes in the DRG, with > 0.1 FPKM. However, to exclude the very weakly expressed genes from our analysis, we set the expression threshold at 1 FPKM, which is a similar threshold to that used in a comparable study [41]. Gene expression at this level can be regarded as reliably detected and is supported by approximately 30 reads which map per 1 kb mRNA, as shown in the Integrative Genomic Viewer (IGV) (Figure S3). Excluding very weakly expressed genes, our analysis revealed the expression of 12984 genes in the TG and 13195 genes in the DRG (> 1 FPKM of all approximately 23000 genes). The expression levels for all investigated approximately 23000 genes can be found in the supplementary data (Table S1). To validate some selected genes, we prepared *in situ* hybridization experiments, for which we used the TG-specific gene Pirt as a positive control (Figure 1A-D).

**Table 1:** Sequencing details of the TG and DRG RNA-Seq experiments.

Sample	Base Pair Sequence Fragments	Total Prepared Reads	Reads with at least one reported alignment (%)	Reads Failing Alignment (%)
Trigeminal Ganglia	36 nt	36807232	31716353 (86.2%)	5090879 (13.8%)
Dorsal Root Ganglia	36 nt	37515634	30227460 (80.6%)	7288174 (19.4%)

### The Superfamily of G-Protein-Coupled Receptors

In the next step, we analyzed the expression patterns for all known non-olfactory GPCRs in mice. A list of 458 GPCRs was established based on several comprehensive studies of murine GPCRs [42–46] (Table S2). Because of the many GPCR genes, we investigated the subfamily of olfactory receptors (OR) separately. 

**Figure 2:**
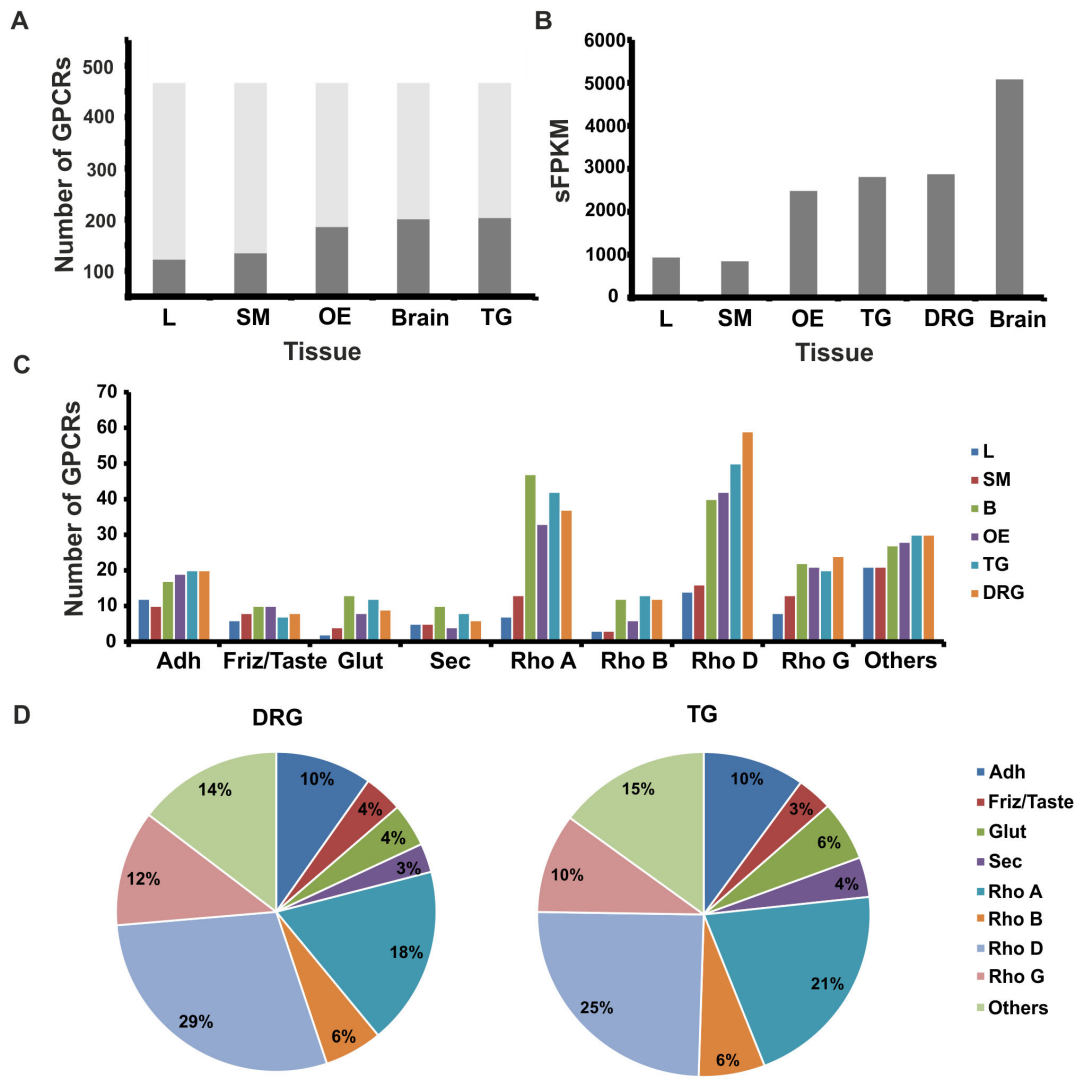
Expression pattern analysis of all detected GPCRs. **A** The bar diagram shows the number of detected GPCRs that had FPKM values higher than 1 for the liver (L), skeletal muscle (SM), olfactory epithelium (OE), dorsal root ganglia (DRG), brain (B), and trigeminal ganglia (TG). Black bars: expressed GPCRs, gray bars: not expressed GPCRs. The lowest number of GPCRs was detected in liver (72) and skeletal muscle (89). The highest count was detected in the TG (197), OE (163), DRG (191), and brain (193). **B** To investigate not only the number of expressed GPCRs but also the general cumulative expression level for each tissue sample, we summarized all FPKM values (sFPKM). The brain (5079 sFPKM), TG (2808 sFPKM) and DRG (2871 sFPKM) had the highest presence of GPCRs. The skeletal muscle had the lowest amount of sFPKM (845), which was followed by the liver (933 sFPKM). **C** The bar diagram shows the expression pattern for all different GPCR subfamilies (secretin (Sec), adhesion (Adh), glutamate (Glut), frizzle and taste (Fzd/Taste), rhodopsin-alpha to -delta (Rho A-D), and not yet classified GPCRs (others). The rhodopsin-delta subfamily (shown without ORs) is expressed at a higher level in the DRG and TG compared with all other tissues. **D** The comparison of the distribution of GPCR subfamilies between the DRG and TG. Most members belong to the rhodopsin-alpha and rhodopsin-delta subfamiles.

In total, the expression of 202 and 204 non-olfactory GPCRs in the TG and DRG, respectively, could be detected with an FPKM that was higher than 1 (Figure 2A). Non-neuronal and non-sensory tissues (liver, muscle) had a significantly lower level of GPCR expression than the neuronal tissues (brain, olfactory epithelium (OE), TG and DRG). The same result can be seen when all FPKM values for the GPCRs > 1 FPKM for each tissue were summarized (sFPKM) (Figure 2B). We analyzed the expression of the distinct GPCR subfamilies in different tissues (Figure 2C). Members of the rhodopsin-delta and adhesion groups show higher expression levels in the TG and DRG than in the other tissues. Rhodopsin, adhesion, and glutamate subfamilies are commonly highly expressed in neuronal tissues. 

The expression patterns for the different GPCR classes in the DRG and TG are highly similar (Figure 2D). Furthermore, nearly 50% of all non-olfactory GPCRs were found to be expressed in the TG and DRG and are mostly rhodopsin-alpha and rhodopsin-delta family members.

### G Protein-Coupled Receptors are Expressed at High Levels in Trigeminal Ganglia

Within the 202 GPCRs that were detected in TG (> 1 FPKM), 106 GPCRs had not been previously described as expressed in the TG, whereas 96 of them were mentioned previously (Table S2). Taking weakly expressed receptors into account (

**Figure 3:**
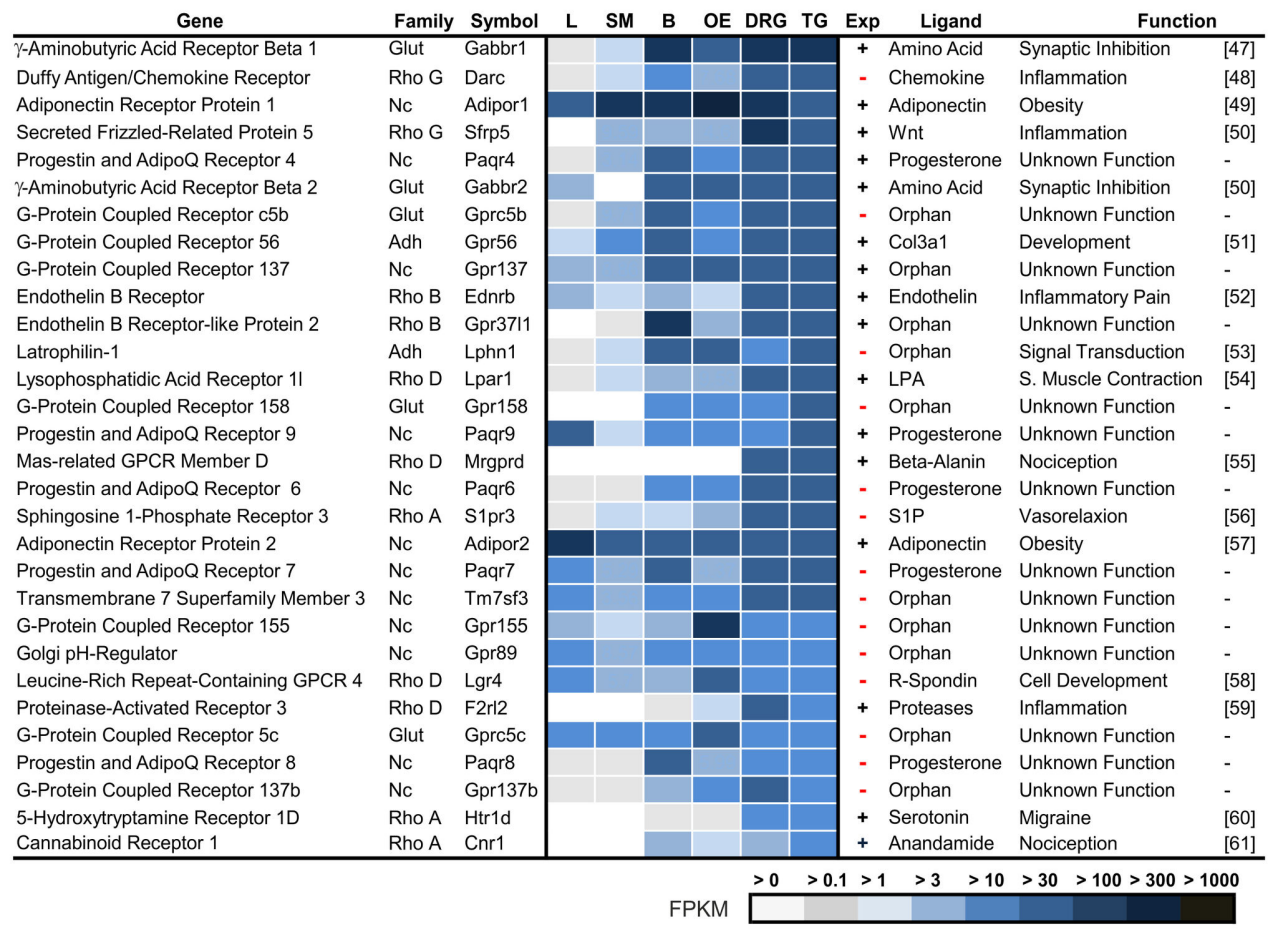
The 30 most highly expressed GPCRs in trigeminal ganglia. GPCRs are listed depending on their expression level in the TG and DRG in comparison with the brain (B), liver (L), olfactory epithelium (OE), and skeletal muscle (SM). The FPKM value, which is an indicator of the expression strength, is represented by the color intensity. Exp describes whether the expression in the TG was previously known (+) or unknown (-). To the best of our knowledge, among the 30 most highly expressed genes, 14 GPCRs have not been previously described as expressed in the TG. Most of the detected GPCRs have unknown functions.

< 1 FPKM), additional 114 GPCRs were detected in the TG, of which 31 were reported previously (Table S2). However, because of this large number of expressed GPCRs, we focused on the 30 most highly expressed GPCRs (Figure 3, Ref. [47–61]). 

Among the most highly expressed 30 GPCR genes in the TG, we detected GPCRs that are known to play a role in nociception, migraine, vasoconstriction, and inflammation. The most highly expressed GPCRs were GABA(B) receptors, endothelin B like Gpr37l1, prostaglandin receptors, and Mas-related receptors (Figure 3). Among the 30 most highly expressed GPCRs, we identified 14 whose trigeminal expression has not been previously described. In total, we newly detected the expression of 107 GPCRs in the TG (Table S2) and additionally list all common GPCR signal transduction proteins (Figure S4). The ligands for several of the most highly expressed GPCRs have not yet been identified. Next, we describe the most prominent of the newly detected GPCRs in TG.

#### Darc

**Figure 4:**
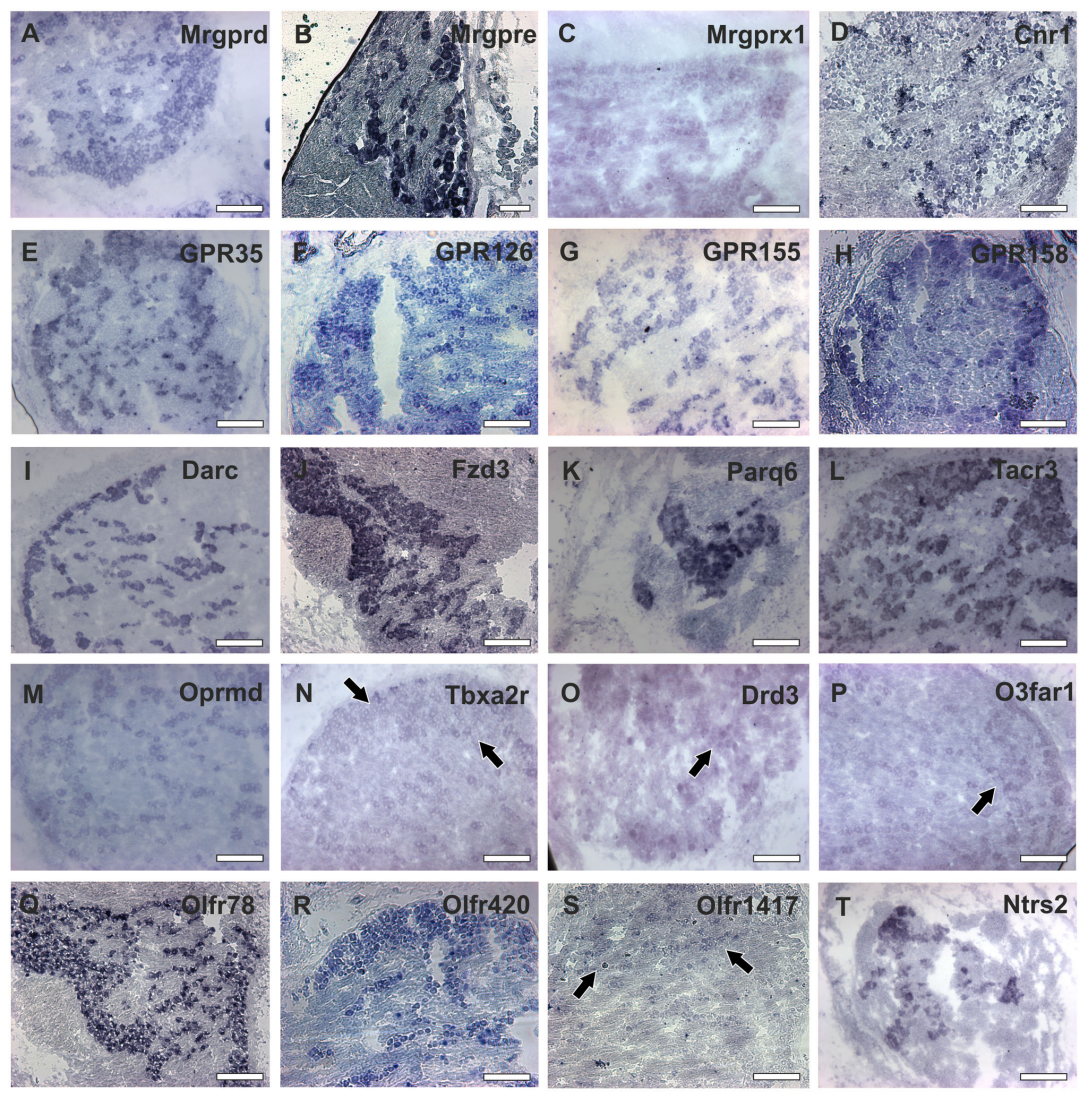
*In* *situ* hybridization experiments in the mouse TG. **A** Mrgprd (Mas-related Receptor D) **B** Mrgpre (Mas-Related Receptor E), **C** Mrgprx1 (Mas-Related Receptor X1) **D** Cnr (Cannabinoid Receptor 1) **E** Gpr35 (G-Protein Coupled Receptor 35) **F** Gpr126 (G-Protein Coupled Receptor 126) **G** Gpr155 (G-Protein Coupled Receptor 155) **H** Gpr158 (G-Protein Coupled Receptor 158) I Darc (Duffy/Antigen/Chemokine Receptor) J: Fzd3 (Frizzled 3) K Paqr (Progestin and AdipoQ Receptors) **L** Tac3 (Tachykainin 3) M Oprmd (mu-Opioid Receptor) **N** Tbx2 (Thromboxane A2 Receptor) **O** Drd3 (Dopamin Receptor D3) **P** O3far1 (Omega 3 Fatty Acid Receptor) **Q** Olfr78 (Olfactory Receptor 78/PSGR) **R** Olfr420 (Olfactory Receptor 420) **S** Olfr1417 (Olfactory Receptor 1417) **T** Ntrs2 (Neurotensin Receptor 2) (Scale for A, C-T 200 µm, B 100 µm).

The duffy antigen/chemokine receptor (Darc) is one of the most highly expressed GPCRs in the TG. The expression of Darc was previously shown for the DRG, basal ganglia, thalamus, and other cortex regions but never for the TG [62]. Darc plays an important role in acute inflammation, infection, and tumor malignancy [48]. We verified the expression of this receptor in the TG by *in situ* hybridization and found this receptor to be strongly expressed at the outermost regions of the TG (Figure 4). In the center of the TG, the expression pattern for Darc was punctate (Figure 4). 

#### Paqr/Adipor

In the TG, six members of the progestin and adipor receptor families (Adipor1-2, Paqr4, 7, 8, 9) are highly expressed; furthermore, several newly detected members of this receptor family show FPKM values of 6-40 (Table S2), which demonstrate that all members of the Paqr family are expressed in nociceptive tissues, such as the TG and DRG. In mammals, the Paqr-family consists of Class I (Adipor1-2, Paqr3) and Class II (Paqr4-9). Class I responds to adiponectin, whereas the ligand for the Class II receptors is progesterone [63–65]. We validated the expression of Paqr6 by *in situ* hybridization (Figure 4) and found that Paqr6 is strongly expressed in all parts of the TG, which correlates well with the detected FPKM of approximately 23. The physiological function of the receptors in the TG is unclear; however, progesterone has shown anti-nociceptive effects in the trigeminal nerve root in a rat LPA-pain model [66]. 

#### S1pr

Furthermore, we could identify the expression of sphingosine1-phosphate receptors (S1pr) in the TG. Meng and colleagues previously showed that S1pr5 is expressed in the TG of embryonic mice [67]. However, our study not only revealed that S1pr3 is the predominant member in the adult TG (Figure 3) but also that S1pr1, -2, and -5 are expressed (Table S2). This class of genes mediates vasodilatation, coordinates angiogenesis with other lysophospholipid receptors, and is known to be involved in developmental processes [56]. 

#### Lgr4

The leucine-rich repeat that contains GPCR is involved in a variety of physiological functions, such as embryonic growth, cell development [68], or in physiological dysfunctions such as in cancer development [69]. Its expression has never been described in the TG and its function is unknown. 

#### Orphan GPCRs

Based on their specific expression pattern in sensory neurons, some of the most highly expressed orphan GPCRs may serve as chemoreceptors. For example, we detected high expression levels of Gpr158, Gprc5b, and Gprc5c in the TG. These three evolutionarily connected receptors belong to the GPCR family C and share a high sequence similarity with GABA(B) receptors, glutamate receptors, and different taste-1 receptors [70,71]. Gpr158 and Gprc5b/c both have a conserved region (pfam00003) that is also found in sweet-taste receptors, which is important for binding sweet-tasting substances, such as cyclamate, or inhibitors, such as lactisole. No specific ligands have been previously described for these receptors. We revealed the expression of Gpr158 in the TG by *in situ* hybridization experiments (Figure 4). Furthermore, highly expressed orphan GPCRs (Gpr155, Gpr126, Gpr137, Gpr137b or Gpr149) could be detected in our RNA-Seq study. Based on their specific expression patterns in the DRG and TG, these orphan GPCRs may be involved in specific functions of the trigeminal sensory system (Table S2). Some of these orphan GPCRs were investigated by *in situ* hybridization experiments (Figure 4).

### G Protein-Coupled Receptors that are Specifically Expressed in Trigeminal Ganglia

**Figure 5:**
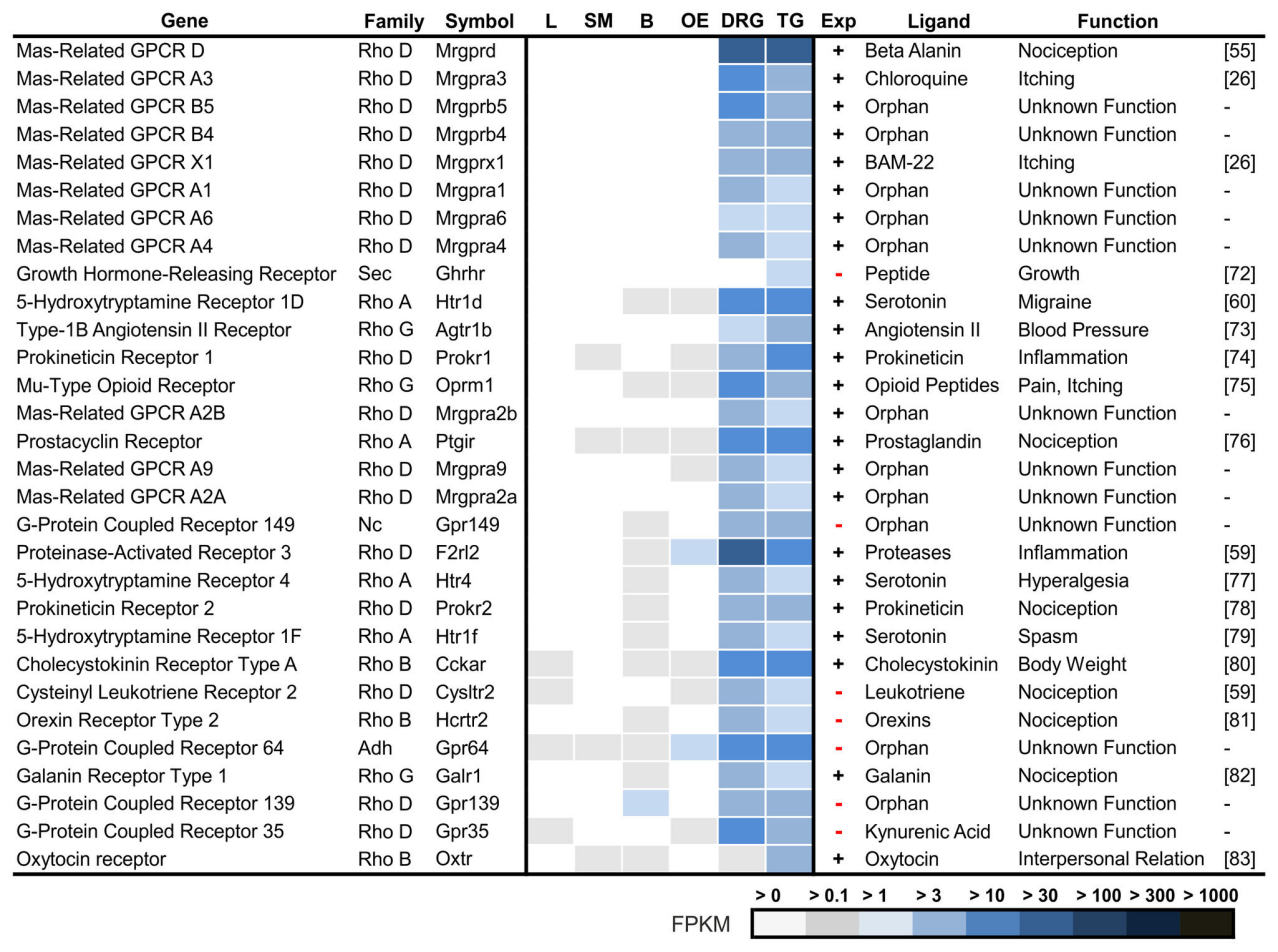
The most TG-specific GPCRs. GPCR genes are ranked according to their specific expression in the TG, which is calculated by the quotient of the FPKM values of TG and the mean FPKM values of brain (B), liver (L), olfactory epithelium (OE), and skeletal muscle (SM). Members of the Mrgprs are the most specific GPCRs that have been detected in the TG and DRG. Among the 30 most specific GPCRs, seven newly detected GPCRs in the TG were identified. Twelve of the most specifically detected GPCRs are still orphans and, based on their specific expression patterns in the TG and DRG, may be important for tissue-specific functions.

To identify the most specific GPCRs for the TG, we calculated a list of genes that are expressed at a higher level in the TG relative to the mean expression in brain, liver, OE, and skeletal muscle (Figure 5, Ref. [55,26,72,60,73–76,59,77–80,59,81–83]). Many of the top 30 candidates that were thereby identified are involved in nociception, migraine, pruritus, inflammation, vasodilatation, and vasoconstriction. 

#### Mrgpr

The most specific and predominantly expressed GPCRs in the TG and DRG belong to the gene family of Mas-related receptors (Mrgpr) [84–86]. The Mrgpr gene family encompasses 20 members, most of which are exclusively expressed in the TG and DRG (Figure S5). Eleven of them belong to the 30 most specific GPCRs for the TG. Mrgpre, Mrgprf, and Mrgprh are also expressed in other tissues (Figure S5). The family of Mrgpr is a relatively newly investigated class of receptors of which only three members have been deorphanized. Mrgprd is involved in β-alanine-mediated pain transmission [55] and influences the perception of mechanical and thermal stimuli [87]. Mrgprx1 and -a3 are receptors for chloroquine and BAM8-22, which induce histamine-independent pruritus [26]. Furthermore, the function for Mrgprb4 was recently shown to be involved in sensing touch or the massage-like stroking of hair [88]. Although the expression of Mrgprs has already been described in several studies, our analysis currently provides the most comprehensive overview for the TG and DRG expression profiles in comparison with various other tissues. Judging by the specific expression of this gene family, our data show that Mrgprs are as specific to the TG and DRG as ORs are to the OE, or pheromone receptors are to the vomeronasal organ (Figure S5). Because of their specific expression pattern, the Mrgpr family may be the most important class of chemo- or somatosensory GPCRs in the TG and DRG. The expression of Mrgprd and Mrgpre was validated by *in situ* hybridization (Figure 4). 

Other well-known TG-specific GPCRs were members of 5-hydroxytryptamine receptors (Htr), the mu-opioid receptor (Oprm1), glutamate receptor 8 (Grm8), the prostacyclin receptor (Ptgir), and prokineticin receptor 1 (Prokr1). Prokr1, which is one of the most specifically expressed GPCRs in the TG is involved in nociception similar to Trpv1. Negri and colleagues showed an impairment of nociception and inflammatory pain sensation in mice that lacked Prokr1 [74]. 

Among the 30 GPCRs that are most specifically expressed in the TG, we identified 7 new candidate transcripts that have not been previously described. 

#### Ghrhr

One of the newly found candidates that is specifically expressed in the TG but not the DRG is the growth hormone-releasing receptor (Ghrhr) (Figure 5). This receptor is expressed in the pituitary gland, and its activation leads to the synthesis of growth hormones. Ghrhr is associated with the growth disease Dwarfism of Sindh [72]. 

#### Hcrtr2

Another interesting candidate transcript is that of the orexin receptor 2 (Hcrtr2/Ox2). Orexin receptors are responsible for sleeping disorders, such as narcolepsy, and can induce sedative effects [89]. In addition to this function, anti-nociceptive effects were reported for Htcr1, but fewer or none were reported for Hcrtr2 [81]. The pathophysiological involvement of Hcrtr2 in pain remains to be investigated. The expression of this receptor in the DRG was recently shown [90]. 

#### Htr

Several 5-hydroxytryptamine receptors (Htr) are, among others, the most specific receptors that are expressed in the TG (Figure 5). We detected the expression of Htr1a, Htr1b, Htr1d, Htr1f, Htr2a, Htr4, Htr5a, Htr5b, and Htr7 (> 1 FPKM). The presence of Htr1a, Htr1b, Htr1d, Htr1f [91,92] Htr2a, and Htr7 [93,94] mRNA in the human TG was detected by PCR. To the best of our knowledge, we could not find any previous reports regarding the expression of Htr4 and Htr5b in the TG, which were weakly expressed with an FPKM of 1-3 (Figure S6). When comparing both types of sensory ganglia, Htr1d expression is 2-fold higher in the TG (~19 FPKM) than in the DRG (~9 FPKM). The expression of serotonin receptors in the TG, cerebral blood vessels, and meningeal tissues is of major interest to understanding the pathophysiology of migraines [95,96].

#### Cysltr2

The expression of the cysteinyl leukotriene receptors 1 and 2 was shown in the spinal cord in rat. Cysltr1 is involved in the development of neuropathic pain [97]. The physiological function of Cysltr2 is unknown; however, Cysltr2 may be involved in cancer progression in other tissues [98].

*Other GPCRs*: Other newly detected TG-specific GPCRs were as follows: Gpr149, Gpr139 and Gpr35. Gpr35 is a recently deorphanized receptor that is activated by agonists such as kuynurenic acid or gallic acid [99,100]. Gpr35 activation causes analgesia in the DRG [100]. Regarding the recently identified ligands, it seems possible that Gpr35 serves as a nociceptor in the TG or as a chemoreceptor. The expression of Gpr35 was validated by *in situ* hybridization in the mouse TG, where we show an expression pattern that is primarily located at the margin of the TG tissue (Figure 4). 

### Other G Protein-Coupled Receptors that are expressed in Trigeminal Ganglia

In addition to the 21 new receptors in the top 30 groups (Figure 3-4), we detected another 79 GPCRs (> 1 FPKM) whose trigeminal expression was not known (Table S2). We surveyed the expression of some of these GPCRs by *in situ* hybridization in TG tissue. Here, we will briefly describe the most interesting newly detected GPCRs as well as some well-characterized GPCRs, which were not among the 30 most specific or highest expressed GPCRs.

#### Tac3

The neuromedin-K receptor (Tac3/Nk3) is expressed in the spinal dorsal horn, the spinal trigeminal nucleus, and several brain regions [101]. Its expression in the TG has never been reported. Tac1 and Tac3 are suggested to be involved in formalin and capsaicin-caused nociception [102,103]. *In situ* hybridization experiments reveal a clear and specific expression of Tac3 in several cells of the TG (Figure 4).

#### Tbxa2r

The thromboxane receptor 2A (Tbxa2r) is a GPCR that we found to be specifically expressed in the TG, which we could also detect by *in situ* hybridization (Figure 4). The expression of Tbxa2r seems to be higher in a subset of trigeminal cells (Figure 4). Tbxa2r is involved in cancer development, anti-platelet aggregation, and vasoconstriction [104–107]. Additionally, Tbxa2r has been suggested to be involved in migraine development (US Patent No: 4.839.384), and new blockers of this receptor may be useful for migraine treatment. 

#### Lgr5

Recent studies have shown that the impairment of Lgr5 is highly up-regulated in various types of cancer cells [108,109]. One study describes how the Lgr5-associated substance fexofenadine induces the relief of symptoms of seasonal allergic rhinitis, including nasal congestion. However, these mechanisms remain unclear [110]. 

#### Cnr

For the cannabinoid receptor 1 (Cnr1), we detected an FPKM value of 12 in the TG. The expression of Cnr1 in medium and large diameter neurons of the TG is well-known [111]. Nevertheless, we confirmed the expression of Cnr1 by *in situ* hybridization experiments (Figure 4). Cnr1 regulates the pre-synaptic inhibition of neurotransmission by reducing the GABA release by GABAergic axons [112]. Cnr1 is coupled to specific types of potassium channels, mobilizes Htr3 receptors, and is negatively coupled to L-type voltage-gated calcium channels (VGCCs) [113–115]. Several previous studies demonstrate the importance of Cnr1 in neuronal anti-inflammatory and nociceptive processes [116,117].

#### Hrh

Histamine receptors (Hrhs) are involved in the perception of pain and in histamine-dependent pruritus [118,119]. Hrhs were detected in the TG by several previous studies, although with different expression patterns [120,121]. In our RNA-Seq analysis, we found that only Hrh3 is expressed at an FPKM value that is higher than 1 (~3 FPKM), which fits well with the expression analysis of Hrh3 in rat embryonic tissues from Hèron and colleagues in 2001 (Table S2) [119]. Hrh1 mRNA in the TG was reported by Kashiba and Senba in 2001 [120] and was found to be weakly expressed in our study (0.9 FPKM). 

#### Other GPCRs

Most of the newly identified GPCRs that are expressed in the TG are orphan receptors, and some of them seem to be specific for the TG and DRG, such as Gpr126 and Gpr149. The expression of Gpr126 was verified by *in situ* hybridization (Figure 4). We confirmed a few weakly expressed genes (0.1- 1 FPKM) with *in situ* hybridization experiments to show that RNA-Seq is able to detect weakly expressed genes such as the dopamine D3 (Drd3) and the fatty-acid receptor omega-3 (O3far). However, due to their low expression in the TG and DRG, it is unclear whether these receptors play any important physiological role in these tissues. 

### Olfactory Receptors

In 1991, Buck and Axel discovered ORs that form the largest superfamily of GPCRs [122]. ORs are primarily expressed in the OE but also in non-olfactory tissues, such as the testes, spermatozoa, prostate, and many other tissues [123–129]. In non-olfactory tissues, these receptors are involved in the proliferation of cancer cells [130] and in the swimming behavior of spermatozoa [127]. In general, ectopically expressed ORs are less represented and less strongly expressed than in the OE [129]. Therefore, we included in our analysis ORs with lower expression levels (> 0.1 FPKM). Of 1125 OR genes, we could detect the expression of 98 ORs in the TG and 33 ORs in the DRG with low abundances (0.1-1 FPKM) (Figure S6). In almost all cases, the expression of ORs was lower than 1 FPKM. In the TG, we found few moderately expressed ORs, such as Olfr920 and Olfr420 with an FPKM higher than 1, that are both expressed exclusively in the TG and OE. We verified the expression of 3 ORs with FPKM levels of 0.1-1 by *in situ* hybridization (Figure 4). The expression of Olfr78, which is a weakly expressed OR (0.2 FPKM) that is also known as PSGR, was validated by our analysis. The human orthologous gene of Olfr78 (PSGR) is a well-known ectopically expressed receptor and evokes calcium responses in the prostate cancer cell line LNCaP and primary prostate epithelium cells that are mediated by steroid hormones (androstenone derivatives). The activation of PSGR inhibits the proliferation of these cancer cells [130]. 

**Figure 6:**
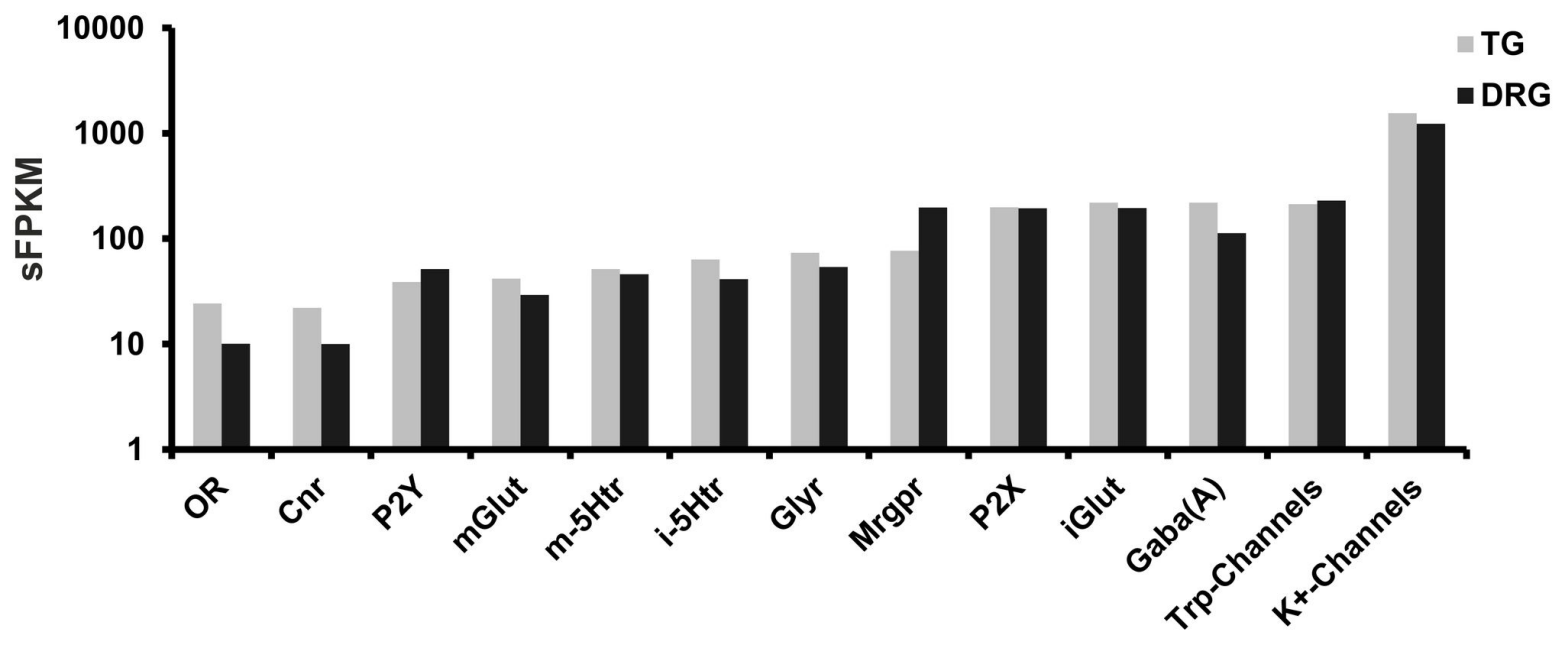
Comparison of cumulative FPKM values (sFPKM). sFPKM values for some prominent ion channels and GPCRs where compared to the sFPKM values of the ORs. The expression of potassium channels is most pronounced in the TG (sFPKM 1551) and DRG (sFPKM 1230), followed by the VGSCs (TG: 963 sFPKM, DRG: 695 sFPKM). Mrgprs, iGlut, P2X, and GABA(A) are expressed at a similar level. Nevertheless, the cumulated FPKM of ORs (TG: 24 sFPKM, DRG: 10 sFPKM) can be compared with the cumulated FPKM of Cnr (TG: 22 sFPKM, DRG: 10 sFPKM), mGlut (TG: 41 sFPKM, DRG: 29 sFPKM), and P2y (TG: 38 sFPKM, DRG: 51 sFPKM). Mrgprs are expressed almost 3-fold higher in the DRG (197 sFPKM) than in the TG (76 sFPKM).

Finally, we compared the cumulated FPKM values for all ORs to investigate not only the number of expressed ORs but also the general cumulative expression level and their presence in TG compared with well-known receptors and channels (Figure 6). Regarding those cumulative values, the presence of ORs (24 sFPKM) is comparable to the cumulative expression of Cnrs (28 sFPKM), P2Y (38 sFPKM), and mGluRs. This high cumulative value argues for the possible functional involvement of the ORs in trigeminal chemosensation. The low FPKM values of individual ectopically expressed ORs may result from a mosaic gene expression pattern, which was also suggested in other studies [129] and was shown for TAARs [131]. It is conceivable that not all TG neurons express ORs and that these receptors might be located only in a few trigeminal neurons, as was shown for Olfr1417 (Figure 4). It is also conceivable that a single TG neuron expresses many different ORs similar to the expression patterns that were shown for bitter taste receptors [132]. The *in situ* hybridization staining patterns for 2 ORs (Olfr78 and Olfr420) argue for the expression of these receptors in a larger number of neurons (Figure 4). Probably all odorants are able to stimulate the TG [133], whereas ORs that are in the TG may support the trigeminal chemosensation of odorants in the nasal mucosa. A detailed table containing the total amount of ORs that are expressed in the TG and DRG can be found in the supplementary data (Figure S7). 

### Ion Channels that are Expressed in Trigeminal Ganglia

Ion channels play an important role in the trigeminal perception of chemical and physical stimuli [134,9,6,135]. We assembled a table of 227 ion channels (potassium channels were analyzed separately) and analyzed their expression in the TG (Table S3). In total, 136 ion channels were detected (> 1 FPKM). Of these ion channels, 103 have already been described in previous studies, whereas the trigeminal expression of 33 ion channels was first detected in the present study. In the class of weakly expressed genes (0.1- 1 FPKM), 24 were known and 27 were new (Table S3). 

### Highest Expressed Ion Channels in Trigeminal Ganglia

**Figure 7:**
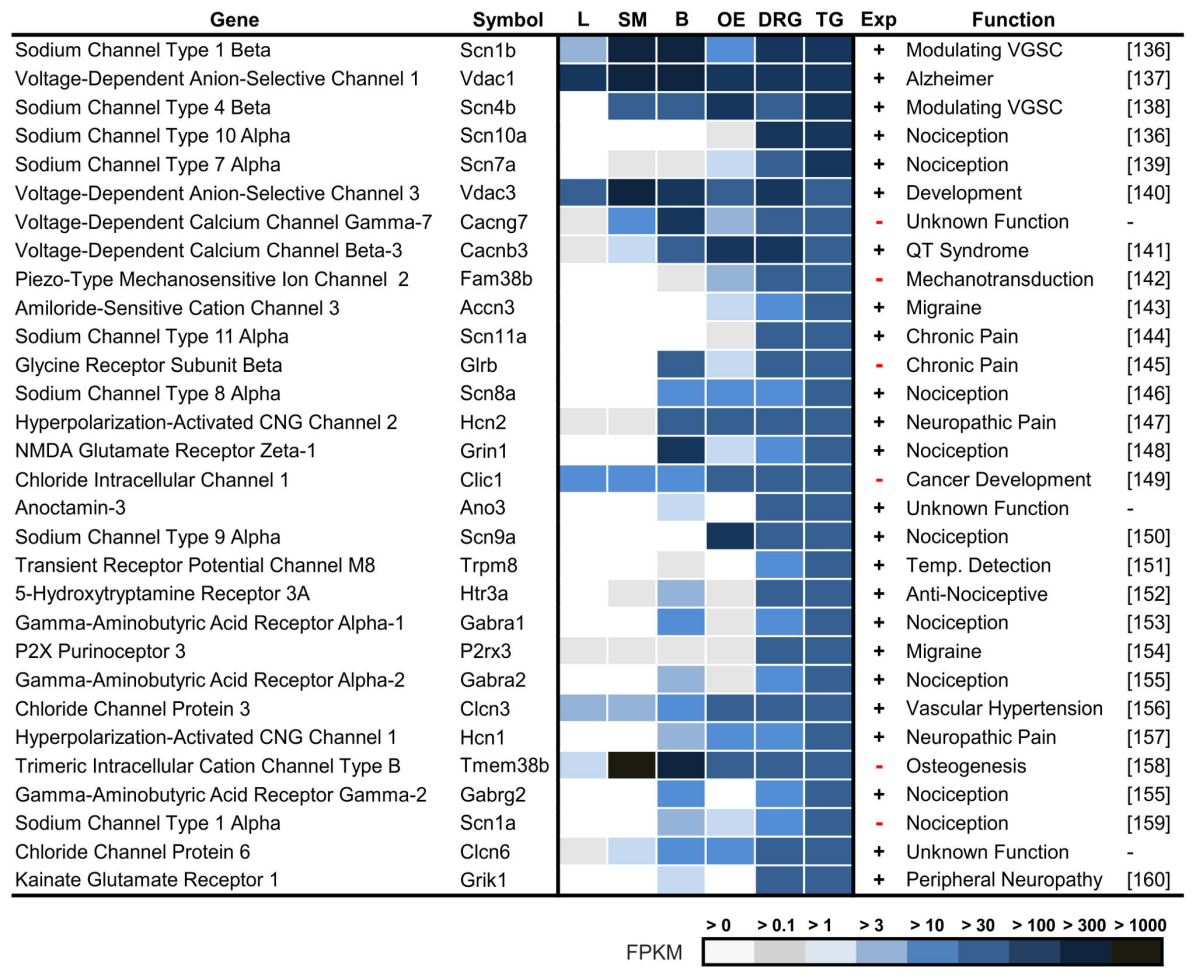
Ranking of the most highly expressed ion channels in the TG. We investigated the expression in the brain (B), liver (L), olfactory epithelium (OE), skeletal muscle (SM), DRG, and TG. Seven of the 30 most highly expressed ion channels have never been described as expressed in sensory ganglia in previous studies (marked with (-)). Many of the most highly expressed ion channels in the TG and DRG are involved in the sensation of pain.

We listed the 30 most highly expressed ion channels for the TG and found 7 new transcripts among these ion channels (Figure 7, Ref. [136–138,136,139–158,155,159,160]). 

#### Ano

**Figure 8:**
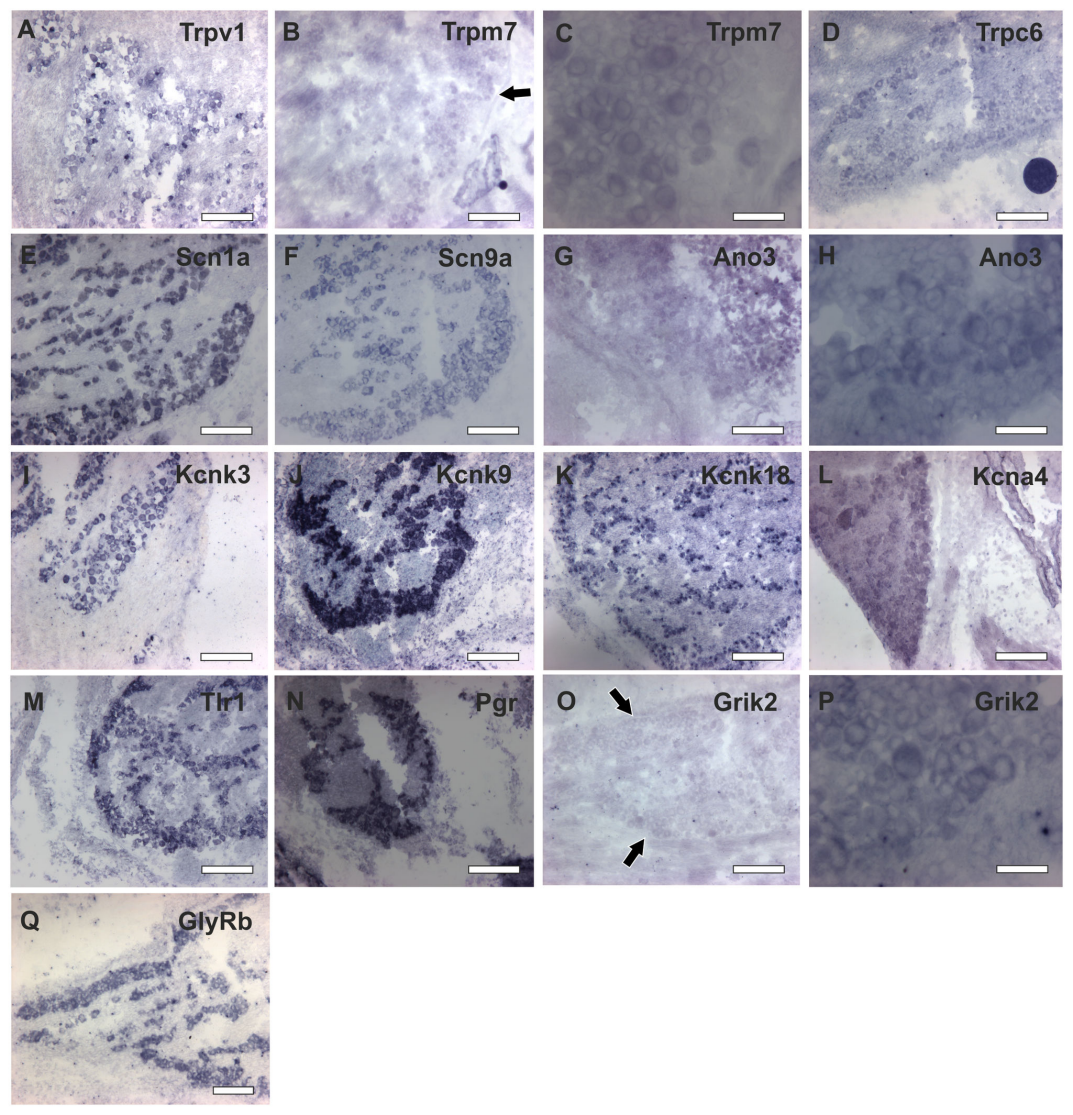
*In* *situ* hybridization for some ion channel in mouse TG. **A** Trpv1 (Transient Receptor Potential Channel 1) **B** Trpm7 (Transient Receptor Potential Channel M7) **C** Trpm8 (Transient Receptor Potential Channel M8) **D** Trpc6 (Transient Receptor Potential Channel C6) **E** Scn1a (Voltage-Gated Sodium Channel 1A) **F** Scn9a (Voltage-Gated Sodium Channel 9A) G GlyRb (Glycine Receptor Beta) **H** Ano3 (Anoctamin 3) **I** Kcnk3 (Potassium Channel K3) **J** Kcnk9 (Potassium Channel K9) **K** Kcnk18 (Potassium Channel 18) **L** Kcna4 (Potassium Channel A4) **M** Grik2 (Glutamate Receptor, Ionotropic, Kainate 2) **N** Pgr (Progesterone Receptor) O Pirt (Phosphoinositide-Interacting Regulator of TRP) **P** Tlr1 (Toll-Like Receptor 1) **Q** Grik2 (Glutamate Receptor, Ionotropic, Kainate 2) **R** Ano3 (Anoctamin 3) **S** Trpm7 (Transient Receptor Potential Channel M7). Weak signals are marked by arrows. Scale for A-P 250 µm, Q-S 75 µm.

One of the most recently detected families of ion channels in the TG is the anoctamin family of proteins (Ano) that are calcium-activated chloride channels with eight transmembrane domains [161]. Ano1 (Tmem16a) and Ano2 (Tmem16b) are expressed in sensory and respiratory tissues of the nose, trigeminal ganglia, septal organ, vomeronasal organ, and Grueneberg ganglion [162]. Ano1 and Ano2 contribute to secretory processes and sensory signal transduction [163–166]. In a recent study, the expression of Ano1, Ano3, Ano4, Ano6, Ano8, and Ano10 was be shown in the TG, some of which may have a role in signal amplification in TG neurons [167]. We validated the expression of the most dominantly expressed Ano3 (52 FPKM) in the TG by *in situ* hybridization (Figure 8). 

#### Scn

The expression of various voltage-gated sodium channel α-subunits (VGSC/Scn/Na_v_) in the TG was already shown in recent studies. The best-characterized channels in peripheral neurons are Scn9a (Na_v_1.7), Scn10a (Na_v_1.8), and Scn11a (Na_v_1.9), which play a role in orofacial pain, trigeminal neuropathic pain, and toothache [168–171]. Scn9a is localized in the axons [172]. A mutation of Scn9a in the OE causes a painful insensitivity or even anosmia [173,174,150,175]. Odorants such as thymol or menthol are able to block VGSC currents as effectively as the local anesthetic lidocaine and thereby prevent nociception [176]. VGSCs are mainly thought to be important in synaptic signaling and in the initiation and propagation of action potentials in neurons [177,178]. In addition to the previously described VGSCs, we detected SCN1a (Na_v_1.1) to be highly and specifically expressed in the TG and DRG (Figure S8). Scn1a can enhance persistent inward sodium currents, and recent studies indicate that a mutation in this gene might play a role in migraine development and in epilepsy [179,180]. We verified the expression of Scn1a and Scn9a by *in situ* hybridization (Figure 8). 

#### GlyR

The glycine beta subunit (GlyRb) is highly expressed in the TG (Figure S9). The presence of GlyRs in neurons of the TG is not yet known. Glycine is the most prominent inhibitory mediator in the whole PNS, and both GABA and glycine are the two best-established inhibitory transmitters. Normally, GlyRb is part of a heteromultimeric complex with GlyR alpha subunits; however, the corresponding alpha subunits are virtually absent in the TG (

< 1 FPKM, Table S3). Potential partners of GlyRs are the GABA(A) receptor subunits (Gabra1/2; Gabrg2), which are highly expressed in the TG (Figure 7 and Figure S9) [167]; however, the existence of such a heteromeric receptor *in vivo* is elusive. GlyRs can modulate chronic and neuropathic pain [181], and a potentiation of GlyRs that are expressed in the spinal cord contributes to the analgesic effects of cannabinoids [182]. The RNA-Seq detection of GlyRb was confirmed in our study by *in situ* hybridization (Figure 8).

#### Tmem38b

The trimeric intracellular cation channel B is highly expressed in the TG. This gene codes for an intracellularly expressed channel that releases calcium from intracellular stores. A dysfunction of this gene in muscle leads to recessive osteogenesis [158].

#### Clic

Chloride intracellular channels (Clics) has been shown to be involved in a variety of chloride ion transports within different types of cellular compartments. Berryman and Bretscher suggest a central role for Clic1, Clic4, and Clic5 in cellular chloride transport [183]. The function of the protein Clic6, which is a novel member of this ion channel class remains elusive [184]. In TG, all members of the Clic channel family could be identified, whereas Clic1 is the most highly expressed subunit (Table S3). Chloride homeostasis plays a crucial role in several functions, which include signal transduction, control of the membrane potential, and the involvement of various secretory and absorptive cellular processes [185].

#### Piezo2

Fam38B (Piezo2) is a mechanically activated cation channel. Coste and colleagues showed the expression of Piezo2 in the DRG and suggested its involvement in mechanically induced sensations such as pain and touch [142,186]. We detected Piezo1 (Table S3) and Piezo2 (Figure 7) in the TG where its physiological role might be the same as in the DRG.

#### Cacng

Voltage-gated calcium channels (VGCCs) are calcium permeable ion channels that are expressed in excitatory cells of muscles and the nervous system. Some members of the voltage-gated calcium channel γ-subunits (Cacng) act as AMPA-receptor regulators in the brain [187]. However, the physiological function of Cacng7 in the TG needs to be investigated, and according to previous made studies, the participation of VGCCs in pain processing is possible [188–190].

### Ion Channels that are Specifically Expressed in the Trigeminal Ganglia

**Figure 9:**
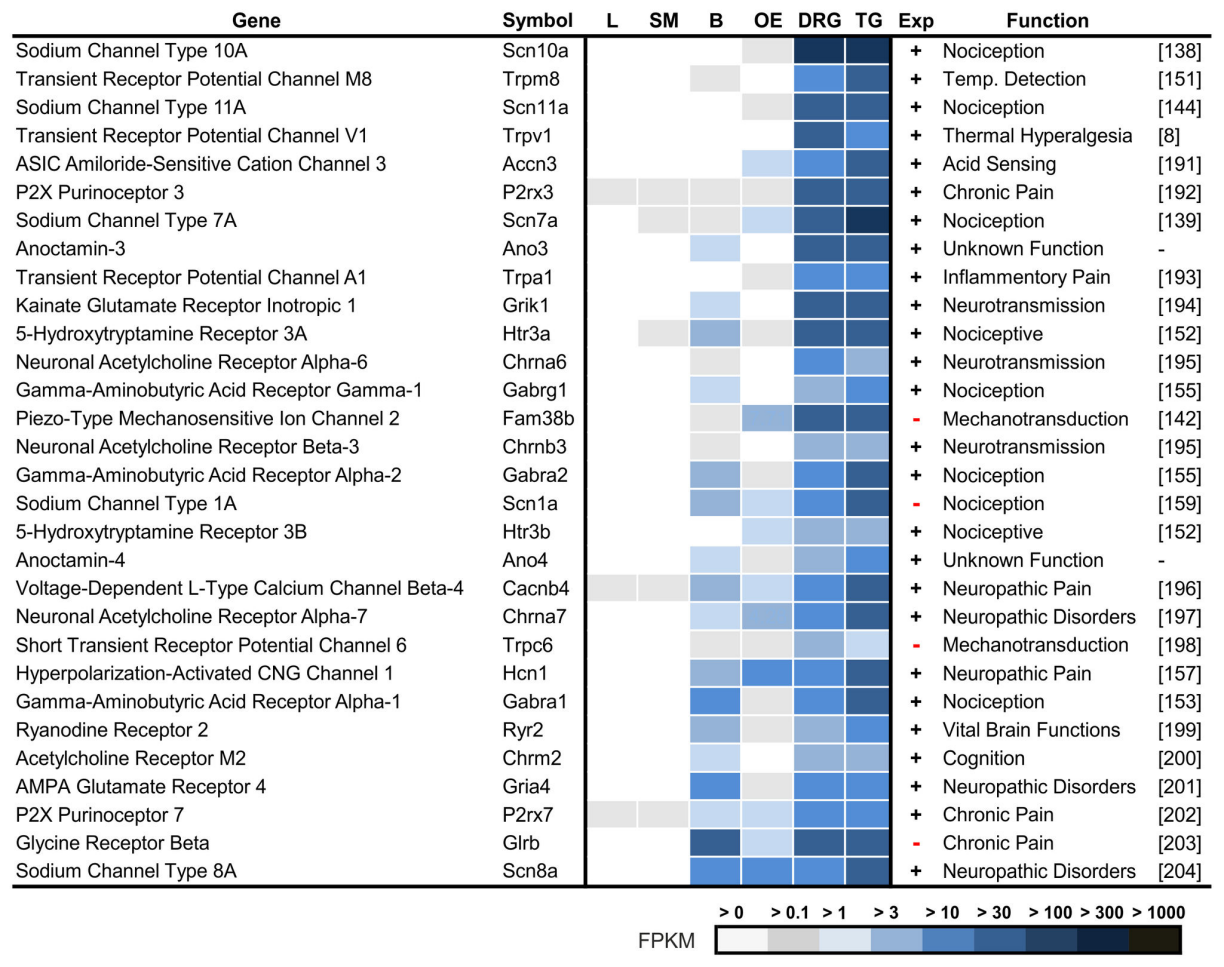
Ranking of TG- and DRG-specific ion channels. We compared the expression levels of the most specific ion channels of the TG and DRG with those in the brain (B), liver (L), olfactory epithelium (OE), and skeletal muscle (SM). Four previously unreported ion channels could be identified among the 30 most specific ion channels in the TG (marked with (-)). Most ion channels are involved in TG thermosensation, mechanosensation and pain perception.

To identify the most specific ion channels for the TG, we calculated a list of genes that are expressed at a higher level in the TG relative to the mean expression levels found in the brain, liver, OE, and skeletal muscle (Figure 9, Ref. [138,151,144,8,191,192,139,193,194,152,195,155,142,195,155,159,152,196–198,157,153,199–204]). Many of the highly expressed ion channel types (Figure 7 and Table S3), such as VGSCs, ionotropic glutamate receptors (iGluRs), purinoreceptors (P2Xs), 5-hydroxytryptamine receptors (Htr3s), Hcn, and GABA(A), also belong to the most specific 30 of the trigeminally expressed ion channels. The most specific ion channels for the TG have been thoroughly investigated, which the exception of the four channels Trpc6, Piezo2, GlyRb, and Scn1a, which have not previously been detected in the TG.

#### iGluR

There are three classes of iGluRs: the α-amino-5-methyl-3-hydroxy-4-isoxazole propionic acid (AMPA) receptors (Gria/Grid), N-methyl-D-aspartate (NMDA) receptors (Grin), and kainite receptors (Grik). iGluRs are expressed in the cell membranes of neurons and are highly concentrated in postsynaptic regions [205]. These channels are involved in signal transduction between neurons, learning, and in a range of neurological dysfunctions [206,207]. iGluRs that are localized in the TG play a fundamental role in processing orofacial pain [208]. The expression pattern of these receptors in the DRG and TG was widely similar (Figure S9). In invertebrates, receptors belonging to the iGluR class respond to several odorants. Furthermore, Benton and colleagues suggest that iGluRs represent a novel class of chemosensory receptors [209]. 

#### P2X

P2X-receptors are ATP-gated cation-permeable ion channels. Seven members (P2X1-7) have been functionally characterized previously [210,211]. In the TG, the expression of P2X2-7 is well-described [212–215]. Our RNA-Seq results show the same expression pattern. Additionally, in our experiments, P2X1 is expressed at low levels (~0.1 FPKM) that are consistent with the low expression of P2X1 in rat TG that was found by Kuroda et al. in 2012 by qPCR [214]. The predominant and specific expression of P2X3 in the sensory ganglia that was revealed by our RNA-Seq data (Figure S9) fits well with the existing immunohistochemical data [212]. P2X-receptors are involved in a wide range of pathophysiological pain mechanics, such as migraine-induced, inflammatory, neuropathic, or acute pain [216,217]. Spehr and colleagues showed that an alteration of the expression of P2X-receptors in rat cultured dissociated trigeminal neurons defines their chemosensory properties [218]. Odors with an aromatic ring structure specifically modulate P2X-receptors in a concentration-dependent manner. Spehr and colleagues suggest that this odor-induced activation of trigeminal neurons could be one of the first steps that contribute to odorant perception by the trigeminal sensory system [218]. 

#### Htr3

Both alpha and beta subunits of inotropic 5-hydroxytryptamine receptors (Htr3a/b) are strongly expressed in the TG, as demonstrated by *in situ* hybridization [219] and by our RNA-Seq data (Figure 7 and Table S3). The most important role of Htr3 in the PNS is the regulation of pain and hyperalgesia that is caused by tissue injury or inflammation [220]. The inhibition of Htr3-evoked currents in cultured trigeminal neurons through synthetic derivates of cannabinoids is discussed as a possible new method of peripheral analgesia [221]. 

#### GABA(A)

In 2006, Hayasaki and coworkers investigated the expression of ionotropic γ- aminobutyric acid receptors (GABA(A)) in rat TG cells [222]. Corresponding with our RNA-Seq data, they detected the expression of the GABA(A) subunits α1-6, β1-3, γ1-3, and δ by RT-PCR (Figure S9). Hayasaki showed a strong immunoreactivity for all GABA(A) subunits in the majority of neurons. The δ and α6 subunits were only observed in small neurons. The most prominently expressed subunits in the TG were α1 and α2 (~50 FPKM), β2 and β3 (~20 FPKM), and γ2 (~44 FPKM). These highly expressed subunits might account for the majority of GABA(A) receptors in the TG. The most common GABA(A) receptor constellation in the CNS is α1 β2 γ2 [223]. GABA(A) receptors are involved in craniovascular nociception, whereas mainly substances such as valproate, allopregnanolone, or propofol may effectively block the neurogenic inflammation that is mediated by GABA(A) receptors [224,225]. GABAergic signaling along with intracellular chloride accumulation plays a critical role in the regulation of signal transmission and pain processed by neurons of the DRG [226–228].

#### Hcn

Hyperpolarization-activated cyclic nucleotide-gated channels (Hcn1-4) are known to be expressed in the TG [229,230]. As in previous studies, our RNA-Seq data revealed that Hcn1-2 are predominately expressed in the TG (40-48 FPKM), whereas Hcn3-4 are expressed at a weaker level (4- 5 FPKM) (Figure S9). Cho and colleagues showed that Hcn4 is mainly present in 9% of all small-diameter TG neurons and in 4.7% of the DRG neurons, consistent with our results (TG: ~4 FPKM, DRG: ~3 FPKM) [230]. The non-selective Hcn cation channels cause an inward cation current and are essential for the maintenance of the neuronal membrane potential. In the PNS, Hcns are involved in several pathoneurological mechanisms such as inflammation-induced pain [231].

#### Trp channels

Transient receptor potential (Trp) channels are possibly the best investigated ion channel subfamily that is expressed in sensory ganglia, and their diverse functions, which include nociception, thermo-, and chemosensation, have been the focus of research in the last few decades [8,232–236]. Trp channels participate in a variety of sensory processes and serve as receptors for environmental and endogenous stimuli and some of them are involved in the signal transduction cascades downstream of metabotropic receptors [237]. In short, 28 members have been described, that fall into six mammalian Trp-subgroups: Trpc (classical-Trp), Trpv (vanilloid-Trp), Trpm (melastatin-Trp), Trpa (ANKTM1-Trp), Trpp (polycystin-Trp), and Trpml (mucolipin-Trp). The best characterized channels that are expressed in the TG and DRG are Trpv1, which senses heat (43°C) and capsaicin, Trpm8, which senses cold (23°C) and menthol, as well as Trpa1, which has been suggested as a sensor of cold (17°C) and isothiocyanates [5,8,134,238–244,151]. In line with previous reports, our analysis confirmed that Trpv1, Trpm8, and Trpa1 are among the 30 most specifically expressed ion channels in the TG (Figure 9). In total, our RNA-Seq detected 16 Trp channels expressed in the TG (Figure S10), which mostly overlap with the most recent RT-PCR study [245], where 17 of the 28 Trp channels were detected and were primarily consistent with our RNA-Seq analysis. Differing from the study of Vandewauw, we detected Trpc2 (1 FPKM), Trpc6 (2 FPKM), and Trpp5 (3 FPKM). As shown in the analysis of Vandewauw, Trp-members, such as Trpv3 (~0.2 FPKM) and Trpv6 (~0.4 FPKM), were also found to be expressed at low levels in our study (Figure S10). Comparing the DRG and TG, we detected differences in the expression levels for Trpc1 (TG: 11 FPKM, DRG: 5 FPKM), Trpm8 (TG: 49 FPKM, DRG: 8 FPKM), and Trpv1 (TG: 12 FPKM, DRG: 29 FPKM). We verified the expression of Trpm7 and Trpc6 in the TG by *in situ* hybridization (Figure 8). 

The Trpc- channel subfamily that was newly detected in the TG, seems to play an important role in somatosensation. In 2009, Staaf et al. showed that the expression of Trpc3, Trpc4, and Trpc5 changes after spared nerve injury of the DRG, suggesting an involvement in nociception [246]. Furthermore, a recent study showed that not only the common Trp channels, such as Trpm8 or Trpa1, are involved in noxious temperature detection but also Trpc5, which can serve as a cold transducer in nociceptive and thermosensory nerve endings [247]. In contrast, Trpc6 is known to play an important role in vasoconstriction [248]. Additionally, these results indicate that the less-studied Trpc channels may be involved in a variety of trigeminal functions. 

### Potassium Channels that are Expressed in Trigeminal Ganglia

**Figure 10:**
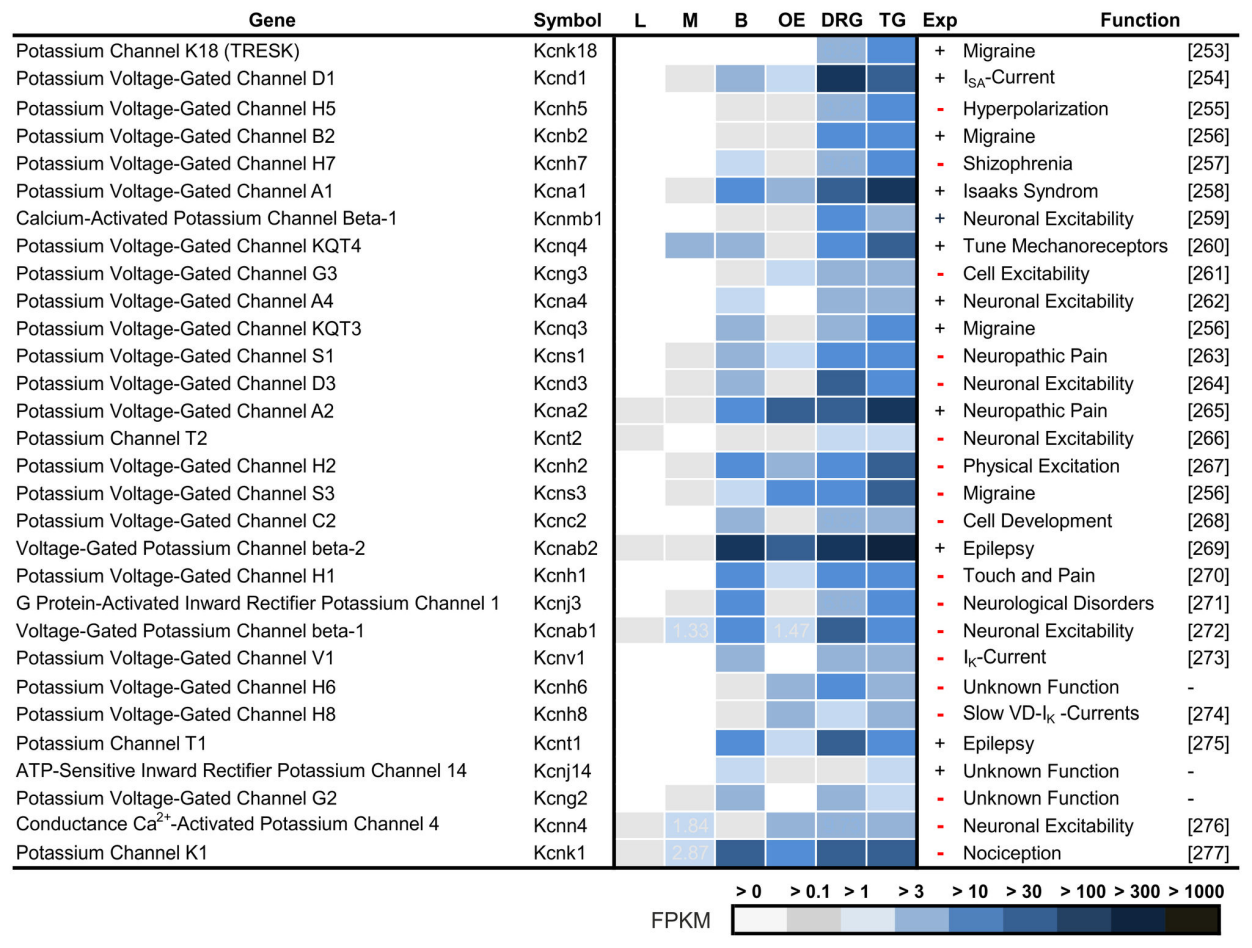
Ranking of potassium channels that are most specifically expressed in the TG and DRG. A comparison of the most specific potassium channels in the TG and DRG compared with the brain (B), liver (L), olfactory epithelium (OE), and skeletal muscle (SM). For the TG, newly detected channels are marked with (-).

In recent years, potassium channels have become a focus of investigation for the mechanisms of somatosensation and nociception [249]. Potassium channels are subgrouped as voltage-gated channels (Kcna-Kcnd, Kcnf-Kcnh, Kcnq and Kcns), calcium-activated (Kcnm-Kcnn), inwardly rectifying (Kcnj), and background/leak, 2 pore channels (Kcnk) [250]. Kcnk channels are a major fundamental determinant for membrane potential and membrane input resistance in excitable cells [251]. Three Kcnk channels (Kcnk3, 9, and 18, also named TASK-1, 3, and TRESK) function as chemoreceptors for hydroxyl-α-sanshool in trigeminal neurons [9], which causes a tingling sensation. Other channels, such as Kcnk2, are heat-activated potassium channels and are important for thermosensation in sensory neurons [252]. We identified Kcnk18 as the most TG-specific potassium channel (Figure 10, Ref. [253–262,256,263–267,256,268–277]). Further, Kcnk18 was the first gene in which a mutation leads to a non-functional channel protein, linked to migraine [278]. To the best of our knowledge, among the 30 most specific potassium channels, 15 of them had not been previously found to be expressed in the TG. Some of these potassium channels, such as Kcns3, are involved in common migraine development processes [256] or are a focus of the therapeutic treatment against diverse neurological diseases and pain, such as Kcnma and Kcnmb, which had already been identified in DRG neurons but not in the TG [279]. 

In summary, only a few potassium channels of the ~80 members have been well investigated to date and are known to play a role in sensing temperature, chemical substances, and pain in the peripheral sensory system [250,280–288,12,289,9]. A detailed list of all potassium channels that are expressed in the TG and DRG can be found in the supplementary data (Figure S11). 

### Other Channels and Proteins that are Expressed in Trigeminal Ganglia

In addition to the 30 most specific or most highly expressed ion channels or GPCRs, we found other genes that are highly or specifically expressed in the TG.

#### Tlr

Toll-like receptors (Tlrs) are a type of pattern recognition receptor and recognize molecules that are shared by pathogens (pathogen-associated molecular patterns) [290–293]. Recent studies have shown that Tlr2 and Tlr4 are also involved in inflammation processes or in chemically induced nociception [294,295]. Eleven members of the Tlr family are known today. In addition to the two known TG-expressed Tlrs, we detected Tlr1, -3, -4, and -5 (Figure S12). The expression of Tlr1 in the TG was confirmed by *in situ* hybridization (Figure 8). 

#### Aqp

Aquaporins (Aqp) are a family of membrane-spanning water channels that are involved in fluid transport [296]. Recent studies indicate the fundamental role of Aqp as a potential therapeutic target for migraines [297]. The water-selective channels Aqp1 and Aqp4 are involved in the pathophysiology of several neurological diseases. Aqp3, Aqp8, and Aqp9 can also transport glycerol or larger solutes. The expression patterns for several Aqps are shown in the supplementary data (Figure S12). 

#### Calm

We found members of the calmodulin (calcium modulated protein: Calm1-3) protein family to be highly expressed (> 500 FPKM) in the TG (Figure S12). Additionally, we found all members and subunits of the calcium/calmodulin-dependent protein kinase types (Camk) in the TG (Figure S12). Calm binds intercellular calcium and alters the signals of different target proteins, which influences signal transmission and neurotransmitter release, as shown for Ano1 in epithelial cells [298]. Camk2a is a well-investigated protein that is important for synaptic plasticity and for the regulation of excitatory synaptic transmission in neurons [299]. It was shown that inhibition of Camk2a in rat TG effectively decreased pain-evoked signals though Trpv1 [300]. Camk2a plays an important role in nociception, inflammation, and injury-evoked events in sensory neurons.

#### Cgrp

It has been suggested that the pathology of migraine relies on the activation of TG nociceptive neurons by the vasodilatation of intracranial extracerebral blood vessels and the subsequent release of vasoactive sensory neuropeptides, most prominently in the calcitonin gene-related peptide Cgrp, which results in an increase in pain [301]. From the trigeminal nuclei, signals are sent to higher centers and pain is perceived. Recently, it was shown that there are two different mechanisms by which Cgrp can induce migraines: the proton-regulated release of Cgrp (with Asic3) and a calcium and synaptosomal-associated pathway (with Trpv1) [302]. In agreement to other studies, we detected a much higher expression of Cgrpα (325 FPKM) and a weaker expression of Cgrpβ (40 FPKM) in the TG [303]. Compared with the TG, Cgrpα-β expression is ~3-fold higher in the DRG (Figure S12). 

#### Pirt

The phosphoinositide-interacting regulator of Trp channels (Pirt) is specifically expressed in sensory ganglia [304]. In all vertebrates, Pirt is a highly conserved membrane protein that binds to PiP2 [305]. In our RNA-Seq analysis, Pirt is highly expressed in the TG and DRG, with an FPKM value of 160 and 174, respectively (Figure S12), but is absent in all other tissues except for the OE (6 FPKM). We used Pirt as a TG-specific marker for *in situ* hybridization experiments (Figure 1 and Figure 8). It is suggested that Pirt plays a fundamental role in many aspects of somatosensation. Pirt is able to interact with different Trp channels and possibly other channels, which indicates a possible regulatory role in neurons [306]. A recent study showed that Pirt is an essential modulator of Trpv1 [307] and Trpm8 function [308]. 

#### Pgr

In recent studies, the expression of the progesterone receptor (Pgr) was shown in the caudal part of the trigeminal nucleus, which is located in the pons [309]. We could show Pgr expression (3 FPKM) in the TG. Pgr is possibly involved in the development of migraines [310] and in anti-nociceptive effects in the DRG of mice [311]. Because the expression of Pgr has never been shown in the TG, we validated our RNA-Seq data by *in situ* hybridization experiments (Figure 8).

#### Nkcc

The sodium-potassium-chloride-cotransporter 1 (Nkcc1, Slc12a2) is a chloride importer that is involved in the regulation of intracellular chloride levels. Nkcc1 is highly expressed in several peripheral sensory tissues and the embryonic CNS. In the embryonic and early postnatal CNS, downregulation of Nkcc1 accompanied by an upregulation of chloride-extruding transporters is linked to the so-called GABA switch that renders GABA-induced signals inhibitory [312,313]. In the olfactory sensory neurons, Nkcc1 maintains high intracellular chloride concentrations crucial for the generation of the receptor potential [314,315]. Our RNA-Seq data revealed the expression of Nkcc1 in the TG (63 FPKM) and DRG (47 FPKM) (Figure S12). Nkcc1 is one of the transporters that is most specifically expressed in the TG and OE. In trigeminal sensory neurons, Nkcc1-mediated intracellular chloride accumulation is crucial for the amplification of capsaicin-induced responses [167]. In the DRG, Nkcc1 activation could be associated to neurite regeneration [316] and Nkcc1 knock-out mice displayed reduced pain sensitivity [317]. In agreement with previous studies, we did not detect Nkcc2 expression in the TG or DRG. 

#### IL

Several members of the interleukin receptor family (IL) were found to be moderately to highly expressed in the TG and DRG. In more detail, the analysis revealed marked expression of IL-1, IL-4, IL-6, IL-10, IL-13, IL-15, IL-17, IL-18, IL-31, and IL-36 receptor subunits. Of special interest are the IL-6 and IL-31 receptors. IL-6 receptor alpha (FPKM 6.7) dimerizes with the promiscuous signal transducer IL-6 receptor beta subunit (= gp130) which we found to be highly expressed in the DRG and TG (FPKM 81.6, 110.6). The heteromeric IL-6 receptor, composed of one IL-6 receptor alpha subunit and two gp130 transducers, mediates the elevation of [Cl^-^]_i_ in DRG neurons via the JAK/STAT pathway in an axotomy model of neurite regeneration [316]. Beyond that, gp130 is required for signaling induced by activation of the IL-6 receptor family member oncostatin regulator beta (OSMR beta) which we found to be expressed in the TG and DRG (FPKM 5, 9.2). Stimulation of OSMR/gp130 was shown to potentiate capsaicin-induced currents in small diameter DRG neurons [318] and appears to be involved in pathological pain processes [319]. 

The IL-31 receptor alpha subunit (FPKM 3.7, 8.8) was found to be highly expressed in human DRG and its ligand IL-31showed marked overexpression in human pruritic atopic skin inflammation samples [320]. In accordance with that, the cytokine IL-31 is associated with pruritus and atopic dermatitis in mice [321]. In the supplementary data we listed the expression profile for all IL members (Figure S12).

### Differential Expression Pattern Comparing Trigeminal Ganglia and Dorsal Root Ganglia

TG and DRG are equally important for the detection of chemicals and the physiology of pain [322]. However, a detailed differential expression analysis of both tissues has never been conducted before. Therefore, the main differences between the TG and DRG were analyzed. 

One main anatomical difference of the TG and DRG is that the TG lacks cell bodies of large-diameter proprioceptors, which rises from the mesencephalic trigeminal nucleus [323,324] and are not included in our RNA-Seq analysis. This could probably be the reason why some classes of GPCRs and ion channels are detected with a higher FPKM in the DRG compared to the TG.

**Figure 11:**
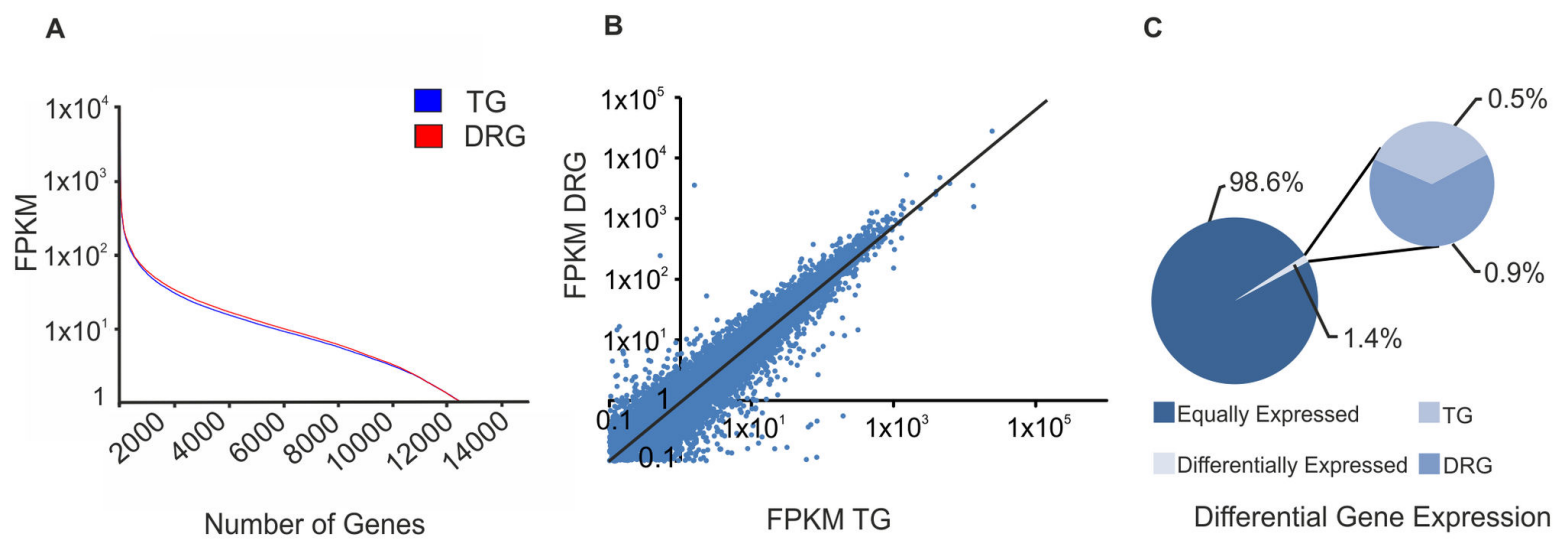
Comparison of expression profiles of the TG and DRG. **A** Differences in the gene expression pattern of the TG and DRG. Of all the genes detected in the TG and DRG, 98.6% were similar (> 1 FPKM), 8113 genes were detected in neither the DRG nor the TG (< 1 FPKM), and 0.5% of the genes were TG- and 0.9% DRG-specific (> 1 FPKM). **B** FPKM distribution for both tissues is highly similar when plotting FPKM values against the number of detected genes. **C** The regression graph visualizes the correlation of the expression patterns for all detected transcripts in the TG and DRG. R^2^= 0.73.

However, the distribution of the FPKM values that were obtained by our RNA-Seq analysis is highly similar between the TG and DRG, and expression patterns for both tissues are highly correlated (R^2^= 0.73, Figure 11A, 11B), whereas the correlation of the TG with other tissues such as the OE is low (R^2^= 0.52) (Figure S13). 

**Figure 12:**
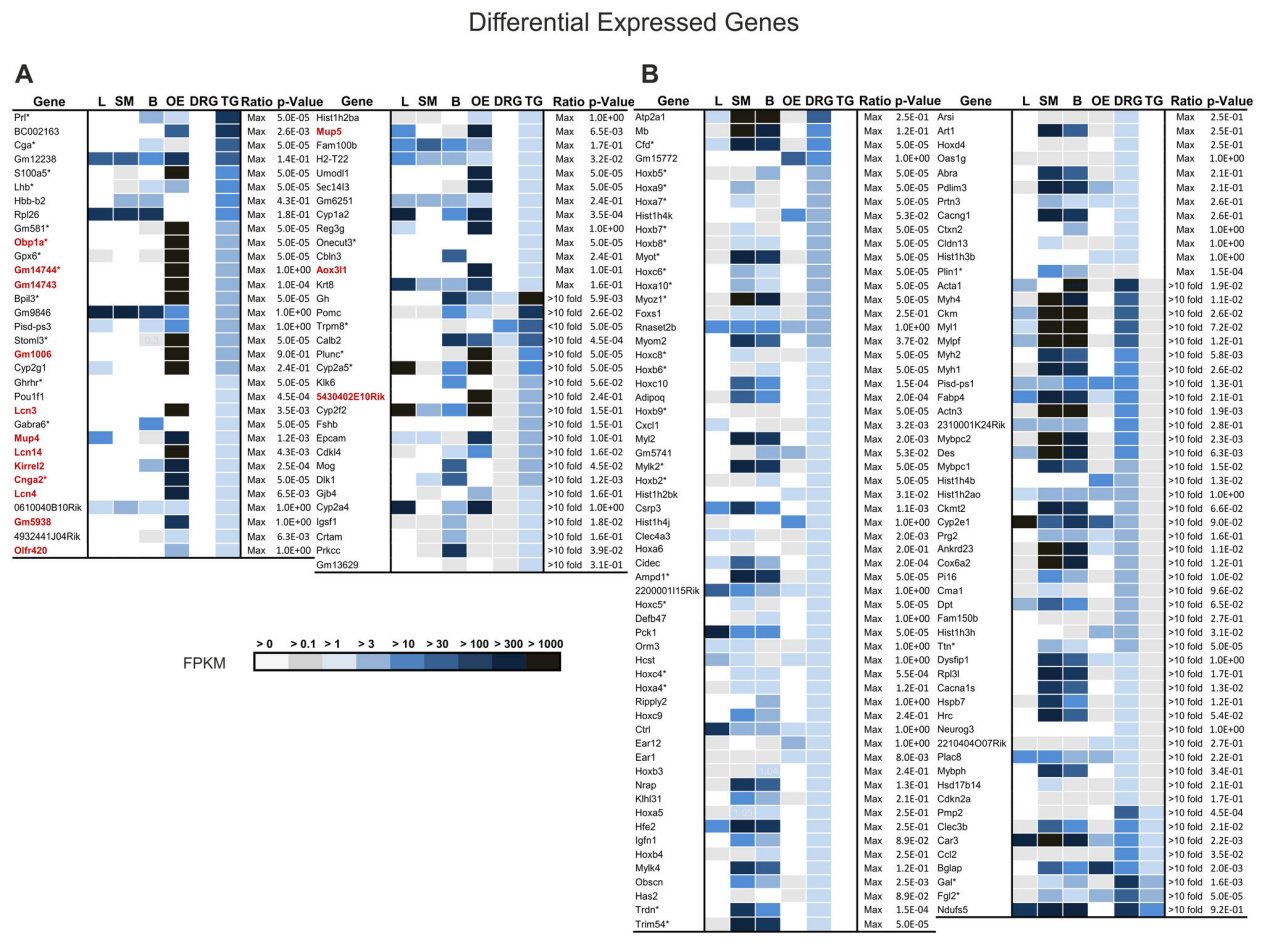
Genes expressed differentially in the TG and DRG. **A** 65 genes are expressed at least 10-fold higher in the TG than in the DRG. Brain (B), liver (L), olfactory epithelium (OE), and skeletal muscle (SM) were used to visualize the global expression patterns for the selected genes. The expression of genes that are marked with (*) is significantly different between the TG and DRG. Interestingly, 15 of the trigeminally-expressed genes are also expressed in the OE and have a function in olfaction. **B** In the DRG, many of the 117 specifically expressed genes play a role in the development or regulation of gene expression, such as Ampd1 or Cfd. Of the 117, 23 genes were significantly expressed at higher levels in the DRG than in the TG. In contrast to the TG, we found several Hox genes with higher expression in the DRG than in the TG.

Our intention was to identify genes with a pronounced differential expression. Therefore, we used Cuffdiff analysis to calculate the amount of significantly differentially expressed genes, and found 19 and 23 genes were significantly higher expressed in the TG and DRG, respectively (Figure 12). Therefore, the relative low number of significantly represented genes is due to the statistically correction for the approximately 23000 parallel comparisons, which makes it difficult to reach a significant level. In addition to the Cuffdiff analysis, we added genes that had at least 10-fold higher differential expression levels, similar to the recently published RNA-Seq analysis of the OE [41] (Figure 12). According to this criterion, the expression of 65 genes is higher in the TG compared with the DRG, and 117 genes have higher expression levels in the DRG compared with the TG (Figure 11C, Figure 12).

We found 12373 genes that were expressed in both tissues with an FPKM > 1 (Figure 11A). Of these genes, 0.5% were detected only in the TG with an FPKM > 1, whereas 0.9% of the genes were only detected in the DRG (

< 1 FPKM).

To gain a functional overview for the gene expression differences we used a gene ontology tool (http://compbio.charite.de) (Table 2). Interestingly, the TG-specific expression reveals a couple of genes (15) that are involved in the chemosensory detection of volatile odorants (marked red in Figure 12). We detected several specifically expressed odorant-binding proteins, such as Lcn3 and Lcn4, Mup3, Mup4, Obp1a, Gm14744, Gm14743, Gm5938, Gm1006, Aox3l1, Gm13629, and 5430402E10Rik, which are primarily expressed only in the OE and TG. Odorant-binding proteins are able to transport hydrophobic molecules through the mucus to receptors [325]. Lcn3 is a putative pheromone-carrier in the vomeronasal organ [326]. The expression of this gene was detected in the nasal septum and in the sensory epithelium of the vomeronasal organ. Miyawaki and colleagues suggested that Lcn3 is involved in the sexual and reproductive behavior of mice [326].

**Table 2:** Classification of differentially expressed genes.

**GO-Terms**	**TG detected**	**DRG detected**	**Gene Functions**
GO:0005549	12	0	Odorant Binding
GO:0004984	1	0	Olfactory Receptor
GO:0050955	1	0	Thermoception
GO:0005179	6	2	Hormone Activity
GO:0032502	12	46	Developmental Process
GO:0005244	1	2	Voltage Gated Ion Channel
GO:0001664	1	2	G-Protein Coupled Receptor Binding
GO:0007154	17	16	Cell Communication
GO:0048878	0	10	Chemical Homeostasis
GO:0042221	10	15	Response to Chemical Stimulus
GO:0007186	12	1	G-Protein Coupled Signaling Pathway

Many of these detected TG-specific transcripts are linked directly into olfactory signal transduction, such as the cyclic nucleotide-gated channel (Cnga2), which is involved in the signal transduction of ORs [327], and the OR Olfr420, which is specifically expressed in the TG and OE. Furthermore, we detected the trigeminal expression of Gα_olf_, which is the olfactory G-protein alpha subunit (Figure S4). The TG- and OE-specific expression of the hemophilic adhesive molecule Kirrel2 has been shown to be impaired in the olfaction signal processing in a Cnga2 knockout mouse [328]. A comparison with the OE transcriptome showed that most of the specific TG transcripts are also highly abundant in the OE; additionally, 15 of the 65 genes are virtually exclusively expressed in both, the OE and TG and are involved in diverse functions of olfaction. 

In contrast, the DRG-specific genes that we found are primarily involved in cellular processes such as cell growth, localization, development, or cell death (71 of 117). Among the 117 genes, 2 voltage-gated calcium channel subunits (Cacna1s and Cacng1) and one GPCR (Cxcl1) were identified. 

## Conclusions

Using the RNA-Seq method, we established a comprehensive analysis of genes that are expressed in the TG and DRG of mice. As we aimed to analyze genes that are involved in sensory processes, we primarily focused our analysis on GPCRs and ion channel expression. 

Our catalog of the most highly or specifically expressed ion channels in the TG demonstrates that nearly all of these ion channels are involved in the sensation of pain or in the detection of chemicals. Although the specific expression and function of most of these channels in trigeminal sensory neurons is well-characterized, we nevertheless identified four new trigeminally-expressed ion channels. In addition, we found many moderately expressed Trp channels whose expression was not detected in the TG before, except in a very recent study [245]. This observation is surprising because the function of Trp channel in the TG and DRG has been in the focus of research for many years. However, a potential function for these newly detected Trp channels in trigeminal sensation is elusive.

We created a detailed list of all potassium channels that are expressed in the TG. In our analysis, we detected the expression of a couple of potassium channels that have not yet been described in the TG. Of the ~80 members of potassium channels, only a few are well investigated, whereas the function of the majority is still unknown.

Little is known about GPCRs expressed in the TG, and many more than we know today may be involved in somatosensation. Interestingly, most of the highly expressed GPCRs that we found in the TG are still orphan receptors. Beyond that, our study revealed the expression of a greater number of GPCRs that are highly expressed in the TG. Our analysis emphasizes the idea that Mrgprs are a family of specifically expressed genes in the sensory ganglia. This receptor type clearly dominates the group of specific GPCRs in the sensory ganglia. Judged by their specific expression pattern, these GPCRs can be best compared with ORs in the OE or to bitter taste receptors (Tas2rs) in the tongue. In contrast to other chemosensory receptor families, much less is known about ligands for the Mrgprs, and only three of the 20 members are deorphanized. Nevertheless, the characterization of the remaining Mrgprs and other orphan GPCRs with potential chemosensory function (e.g., Gprc5b, Gprc5c, Gpr178, or Gpr158) is a prerequisite to further our understanding of the sensory functions of the DRG and TG. Our RNA-Seq study may help to identify the important candidates that will be the basis of future studies. 

Differences in tissue-dependent sensory functions are correlated with differences in the expression patterns for genes that code for membrane receptors. A differential transcriptome analysis of the TG and DRG identified several genes with pronounced expression variances. Several of the genes that are specific for the TG are also highly expressed in the OE and are involved in the chemical detection of odorants. This observation implies that the TG has a better capacity than the DRG to detect chemical cues. Similarly, the higher cumulative FPKM values for ORs in the TG and for Mrgprs in the DRG strongly argue for a more chemosensory or somatosensory specialization of these two sensory systems, respectively.

In general, a detailed expression profile of all genes can be an important tool to promote our understanding of the function of the TG and DRG. In particular, the analysis of GPCRs and ion channels helps to identify new candidates that participate in chemical detection or nociception. This analysis generates a basis for comparison, aims to encourage further studies on ion channels and GPCRs that are expressed in the TG and DRG, and sheds light on the main differences between these functionally and anatomically similar structures.

## Materials and Methods

### Animals

All experiments involving animals were carried out in accordance with The European Union Community Council guidelines and approved by the competent state office of the Federal Land of Northrhine Westphalia (file 87-51.04.2010.A180) and the German Tierschutzgesetz (law on animal protection). Adult male CD1 mice were obtained from Charles River (Sulzfeld, Germany). 

### Excision of the TG and DRG

For preparation of the TG, mice were sacrificed, sculls were opened, the brains removed, and the exposed TG were dissected using forceps. The ganglia were washed in PBS and then further processed for RNA extraction as described below. For preparation of the DRG, the same animals were used. The spines were opened along the midline by cutting through the vertebral canal and the spinal marrow was removed to expose the DRG located in between the vertebral bodies. The thoracaic, lumbal, and sacral DRG were removed, washed in PBS, and then further processed for RNA extraction.

### RNA Isolation and Next Generation Sequencing

RNA from murine DRG or TG of pooled tissues from 8 male adult CD1 mice was isolated with the RNeasy Mini Kit (Qiagen, Hilden, Germany) according to the manufacturer´s protocol, which included DNaseI digestion. At the Cologne Center for Genomics NGS unit, libraries for sequencing were constructed from the total RNA and were subjected to DSN normalization. RNA-Seq was performed on the Illumina GA_IIx_ sequencing platform with a 36-nucleotide length. We essentially analyzed the sequence data as described previously [36]. The raw sequence data were aligned to the mouse genome reference sequence (mm9) using the TopHat aligner. To avoid the alignment of highly repetitive reads, a multiread-correction was used, which allowed up to 5 hits per read.

Consequently, we could map 33 million or 32 million reads for the TG or DRG. Output BAM-files were sorted and indexed using the SAM tools software package [329]. FPKM values were subsequently calculated by the Cufflinks program using the mm9 RefSeq reference transcriptome. We further used a masked command M and the mask GTF rmsk.gtf to hide all possible reads that were RNA repeats, including tRNA, rRNA, snRNA, scRNA, and sprRNA, short as well as long interspersed nuclear elements (SINE, LINE), and other different classes of repeats. In order to investigate the expression differences between the TG and DRG, we used Cuffdiff with the common RefSeq reference transcriptome. 

Schöbel and colleagues already presented a small subset of our generated data, which describe the expression of Ano1-10 and Ttyh1-3 channels in the TG [167]. 

For comparison, we reanalyzed the already-published raw RNA-Seq data from the brain, liver and skeletal muscle in the same manner as our own data. The data sets were available in the NCBI SRA archive and the following accession number: mouse brain (SRR006488, SRR006489), mouse liver (SRR006490, SRR006491, SRR001360, SRR001359) and mouse skeletal muscle (SRR001361, SRR001362, SRR006492) [38]. The transcriptome from the OE of 4-week-old CD1 mice was calculated using 37 million (male) or 52 million (female) 36 bp that were reads generated by Illumina sequencing on a GA_IIx_ platform. The analysis of the pooled OE was performed with the same parameters that were used for the TG and DRG. A detailed analysis on the OE transcriptome will be presented else-where.

The identification of genes that were enriched in the TG and DRG was essentially performed as described in previous RNA-Seq comparison studies for the OE [41,330]. We focused only on protein coding genes that were expressed with at least 1 FPKM and at least 10-fold difference.

### Calculation of GO-terms

 Go-Terms were calculated with a free online tool that is available at http://compbio.charite.de/contao/index.php/ontologizer2.html. 

### In Situ Hybridization

Digoxigenin-labeled sense and antisense RNA-probe fragments, which were typically approximately 200-500 nucleotides in length, were generated from cDNA fragments cloned into pCDNA3 (Invitrogen, Freiburg, Germany) by *in vitro* transcription that was performed with the DIG RNA labeling mix (Roche, Palo, Alti, CA) and T7 or SP6 RNA polymerase according to the manufacturer’s instructions. The primer pairs used were as follows:

Ano3:

forward: GCATATGAATTCCTTTGGTGAGAAGATTGGCTTA,

reverse: GCATATGCGGCCGCTTGGCTTTCGTTCATTGTGA;

Cnr1:

forward: GCATATGAATTCGCTTGCGATCATGGTGTATG,

reverse: GCATATGCGGCCGCGTGTTATTGGCGTGCTTGTG;

Darc:

forward: GCATATGAATTCCAAGGGGCTGAAGATAGCAC,

reverse: GCATATGCGGCCGCGTAGCCACACAGTGCAGCAT;

Drd3:

forward: GCATATGAATTCGAGCACATAGAAGACAAACCATATC,

reverse: GCATATGCGGCCGCCGAGCACAATGACCACCAT;

Fzd3:

forward: GCATATGAATTCGCCACCATGTCCCAATATGT,

reverse: GCATATGCGGCCGCCTACTCGGTCCTCCAGCAAA;

GlyRb:

forward: GCATATGAATTCGCATCTTCTCCGTGCTCAGT,

reverse: GCATATGCGGCCGCCTGCAAAGTGCTGATATGAAC;

Gpr35:

forward: GCATATGAATTCCTGCTTCCGTCAACAACTTCT,

reverse: GCATATGCGGCCGCGCCCTGCAAAGAGCAGAAGACC;

Gpr126:

forward: GCATATGAATTCCCAAAGTTGGCAATGAAGGT,

reverse: GCATATGCGGCCGCCAATGGAGCCCCAAGAATTA;

Gpr155:

forward: GCATATGAATTCTGGGACTTGGATTTCTACGC;

reverse: GCATATGCGGCCGCTCAGTCGCCTGATTTTTCCT;

Gpr158:

forward: GCATATGAATTCCCTTTCACGAACAGCACAAA,

reverse: GCATATGCGGCCGCTGATCAGATGTTTGCCCTTG;

Grik2:

forward: GCATATGAATTCCACATTCAGACTCGCTGGAA,

reverse: GCATATGCGGCCGCGTCTTCGTACACCACCGTCA;

Kcnka4:

forward: GCATATGAATTCAGGTGGTTCTGAGGAGAGTGAG,

reverse: GCATATGCGGCCGCGGGCTTCGGCATAGAAAGTA;

Kcnk3:

forward: GCATATGAATTCGATCGTGAGGTACCTGCTGCAC,

reverse: GCATATGCGGCCGCGATGTGTCGGACGTGGAGAGGT;

Kcnk9:

forward: GCATATGAATTCGATTATATCCTGGTGGGCCTGAC,

reverse: GCATATGCGGCCGCGATAAAACGGACCGGAAGTAGGT;

Kcnk18:

forward: GCATATGAATTCGATACCAGGCTCGGTAAGTTCCT,

reverse: GCATATGCGGCCGCGATGGTGGTCAGTGTCACAAAGC;

Mrgprd:

forward: GCATATGAATTCGCAGAGGTCTCCCTTCTGTG,

reverse: GCATATGCGGCCGCTACCAGATGGGGAAAAGCAC;

Mrgpre:

forward: GCATATGAATTCCAGGGAGAAATGGCTTTCAA,

reverse: GCATATGCGGCCGCTTCAGGGAAGTTCAGCTGGT;

Mrgprx1:

forward: GCTATGAATTCTCGCTCTCACAGTGATGGC,

reverse: GCATATGCGGCCGCTGTCCTCCAGAGCCCTCTTA;

Ntrsr2:

forward: GCATATGAATTCGTGAACGTGCTGGTCTCCTT,

reverse: GCATATGCGGCCGCGCCCCAGGGAGAGGGTCTTTCT;

O3far1:

forward: GCATATGAATTCACTTCCCTTTCTTCTCGGATG,

reverse: GCATATGCGGCCGCCGAGTAACCCCATATGAAAGC;

Olfr78:

forward: GCATATGAATTCGGTGGCTCTGGTCCGGGGAT,

reverse: GCATATGCGGCCGCGCCACAGGAGGCAGCAGCAGGT;

Olfr420:

forward: GCATATGAATTCCCCAGCTGACCCTCGGTTGC,

reverse: GCATATGCGGCCGCGCTCAGGTGCGAGACGCACGTGGAAA;

Olfr1417:

forward: GCATATGAATTCGGCCATCTGTCACCCTCTGCG,

reverse: GCATATGCGGCCGCGCAAGCAGGAGGAAGCTTATCACCACC;

Oprd:

forward: GCATATGAATTCAGACCGCCACCAACATCTAC,

reverse: GCATATGCGGCCGCCTTTGACAGGATGGCAGACA;

Paqr6:

forward: GCATATGAATTCGCCACCAGCTGTTCCATATC,

reverse: GCATATGCGGCCGCGCAAGGAGGCCGTGAAAGCAG;

Pgr:

forward: GCATATGAATTCTTCCTTTGGAAGGACTGAGG,

reverse: GCATATGCGGCCGCGCATCATGCAAGCTGTCGAGAA;

Scn1a:

forward: GCATATGAATTCGATCCAGTCGGTGAAGAAGC,

reverse: GCATATGCGGCCGCTCCAGTCAAACTCGAACACG;

Scn9a:

forward: GCATATGAATTCGCCCTGATCCAATCAGTGAA,

reverse: GCATATGCGGCCGCTCTAATGTTTCATTCTGCTCAAGG;

Tac3:

forward: GCATATGAATTCGGGTCCCATACAGGGAATCT,

reverse: GCATATGCGGCCGCGGGCCAAGATGATCCAAATA;

Tbxa2r:

forward: GCATATGAATTCTGGTTCGCTGCGTCCTTT,

reverse: GCATATGCGGCCGCAGAAGGGCCGTGTGATGC;

Tlr1:

forward: GCATATGAATTCATGACTAAACCAAATTCCCTCATC,

reverse: GCATATGCGGCCGCGCGAAGAGATTCGGGGTCTTCTTT;

Trpc6:

forward: GCATATGAATTCGCATGATATGGGCTGAATGT,

reverse: GCATATGCGGCCGCGCCCAGATTGTAGTATTTAACGTTGTCC;

Trpm7:

forward: GCATATGAATTCAGTGGAGCAGATGAGCATTC,

reverse: GCATATGCGGCCGCGCAAATCTTGTCCAAACAGATTATATTG;

Trpm8:

forward: GCATATGAATTCGCCATCAACACCTCTGTCAA,

reverse: GCATATGCGGCCGCCCATTTGATCCAGCTCTCAA;

*In situ* hybridization experiments of the TG were performed with male and female P7 CLB6 mice. The mouse brains were fixed overnight in 4% paraformaldehyde in PBS at 4°C (7.5 pH). The next day, the brains were incubated in 10% and 20% sucrose for 1 h each and additionally overnight in 30% sucrose. The mandibular and the frontal part of the nose were removed. Afterwards, transversal sections (14 µm) of quickly frozen heads, which were embedded in the tissue freezing medium OCT that supports tissue during cryotomy (Leica Microsystems, Bensheim, Germany), were cut on a cryostat (Leica Microsystems, CM 3050S, Bensheim, Germany) and mounted on Superfrost® Plus Slides (Menzel-Gläser, Braunschweig, Germany). After dehydration using an increasing ethanol series, slices were stored at -80°C until further use. *In situ* hybridizations were performed as described with minor modifications [74]. Briefly, fixed cryosections were incubated in RIPA-buffer, followed by an acetylation step with acetic anhydride in TEA buffer. Next, a prehybridization step in 50% deionized formamide, 10% dextran sulfate, 5x Denhardts solution, 5x SSC, 10 mM DTT, 250 µg/ml yeast tRNA 500 µg/ml sheared and denatured herring sperm, 50 µg/ml heparin, 2.5 mM EDTA, and 0.1% (v/v) Tween-20 was carried out for 1 h at 55°C to prevent the nonspecific binding of riboprobes. Each incubation step was followed by wash step with SSC or PBST -buffers. 

Finally, 50 ng antisense riboprobes was hybridized at 55-65°C on cryosections that were mounted on slides overnight. The hybridized mRNA was visualized using an alkaline phosphatase-conjugated antibody to digoxigenin and the hydrolysis of nitro-blue tetrazolium chloride/5-bromo-4-chloro-3-indolylphosphate p-toluidine. An antisense and a control sense probe were tested in parallel. The slides were covered with cover slips using polyvinyl-alcohol that contained embedding medium (Mowiol®, Immo-Mount, Thermo-Scientific, Braunschweig, Germany). Digital images were obtained with an Axiocam camera on an Axioscope2 microscope (Zeiss, Oberkochen, Germany). All images of sense and antisense samples were recorded under the same conditions (brightness contrast and light exposure time).

A signal was considered positive when the antisense labeling was noticeably darker visually than the sense labeling. We used Pirt as a positive control, which is highly and specifically expressed in the TG.

## Supporting Information

Figure S1 **Expression strength for housekeeping genes in all analyzed tissues.** Expression analysis for known expressed housekeeping genes. To show that our calculated FPKM values for all tissues is comparable in principle, we analyzed the expression of known expressed housekeeping genes, which could be detected in all of our tested samples.
(TIF)

Figure S2 **Distribution of FPKM values compared with the different tissues used.** The highest numbers of genes are expressed between 1-10 FPKM in all tissues. There are fewer highly expressed genes with an FPKM of > 100.
(TIF)

Figure S3 **Integrative Genomic Viewer.** Mapped reads for 1-kb large genes, which are expressed with 0.1 FPKM, 1 FPKM, and 10 FPKM.
(TIF)

Figure S4 **Expression profile for all common GPCR signal transduction proteins.**
(XLSX)

Figure S5 **Expression profile for the TG and DRG specific family Mrpgrs.**
(XLSX)

Figure S6 **Expression profile for the Htr family.**
(XLSX)

Figure S7 **Expression profile for all existing ORs in the TG and DRG.**
(XLSX)

Figure S8 **Expression profile for all members of the VGSCs.**
(XLSX)

Figure S9 **Expression profile for GlyRs, GABA(A), iGluts, P2Xs, and Hcns.**
(XLSX)

Figure S10 **Expression profile for all existing Trp channels in the TG and DRG.**
(XLSX)

Figure S11 **Expression profile for all existing potassium channels and subunits in the TG and DRG.**
(XLSX)

Figure S12 **Expression profile for other interesting membrane proteins.**
(XLSX)

Figure S13 **Comparison of the gene expression between the OE and TG and the OE and DRG.**
(TIF)

Table S1 **Table with the expression pattern for all analyzed ~23000 genes.**
(XLSX)

Table S2 **Table with the expression pattern for all known GPCRs, sorted by their subfamily and marked whether they are known to be expressed in the TG.**
(XLSX)

Table S3 **Table with the expression pattern for all ion channels in the TG and DRG.**
(XLSX)
