# Supplementary figures and images for: Comprehensive RNA-Seq Expression Analysis of Sensory Ganglia with a Focus on Ion Channels and GPCRs in Trigeminal Ganglia
Source: PLoS One. 2013 Nov 8;8(11):e79523. doi: 10.1371/journal.pone.0079523 (PMC3832644; doi:10.1371/journal.pone.0079523)

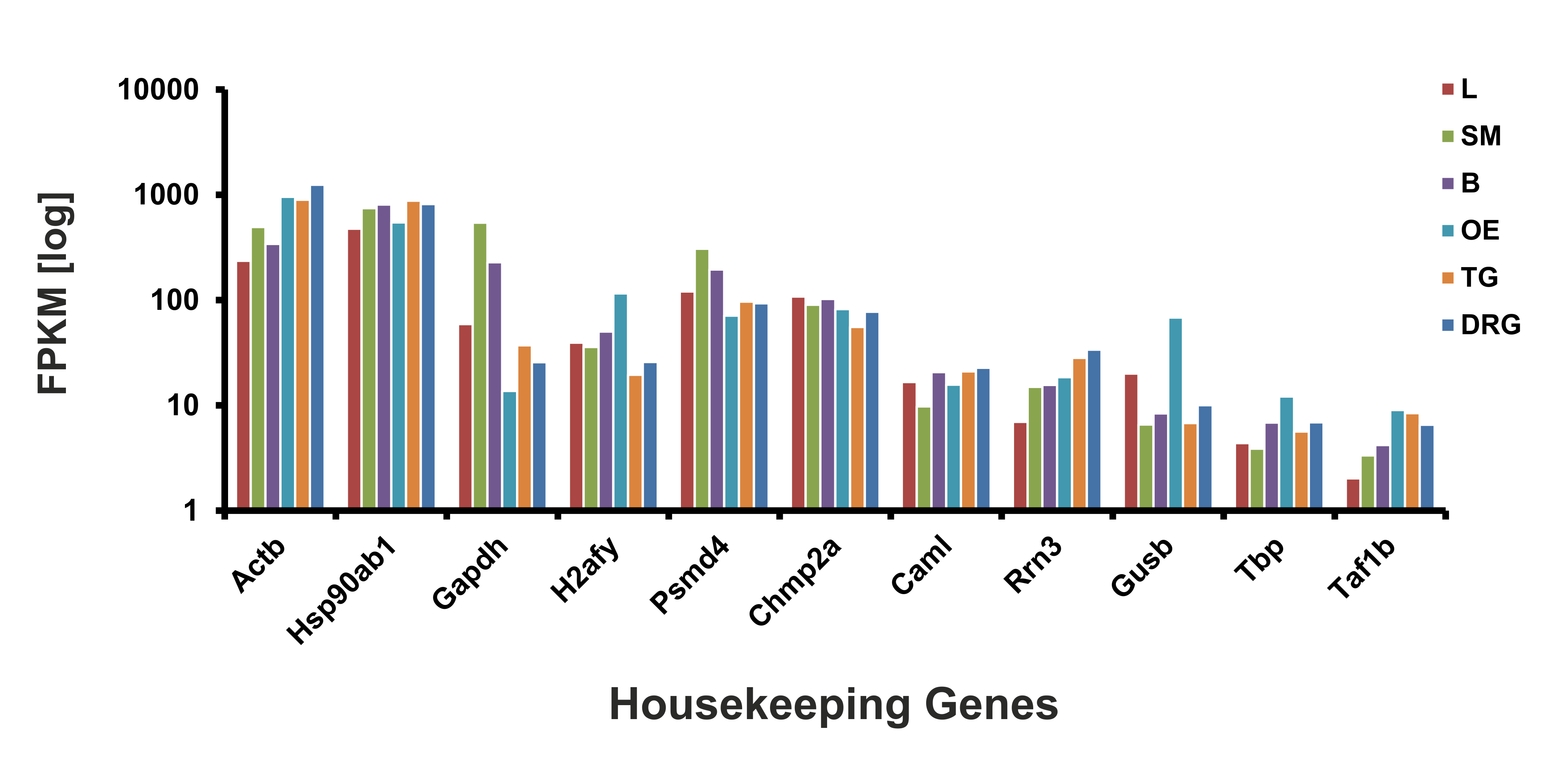

Supplement: Figure S1 — Expression strength for housekeeping genes in all analyzed tissues. Expression analysis for known expressed housekeeping genes. To show that our calculated FPKM values for all tissues is comparable in principle, we analyzed the expression of known expressed housekeeping genes, which could be detected in all of our tested samples. (TIF) [file pone.0079523.s001.tif]

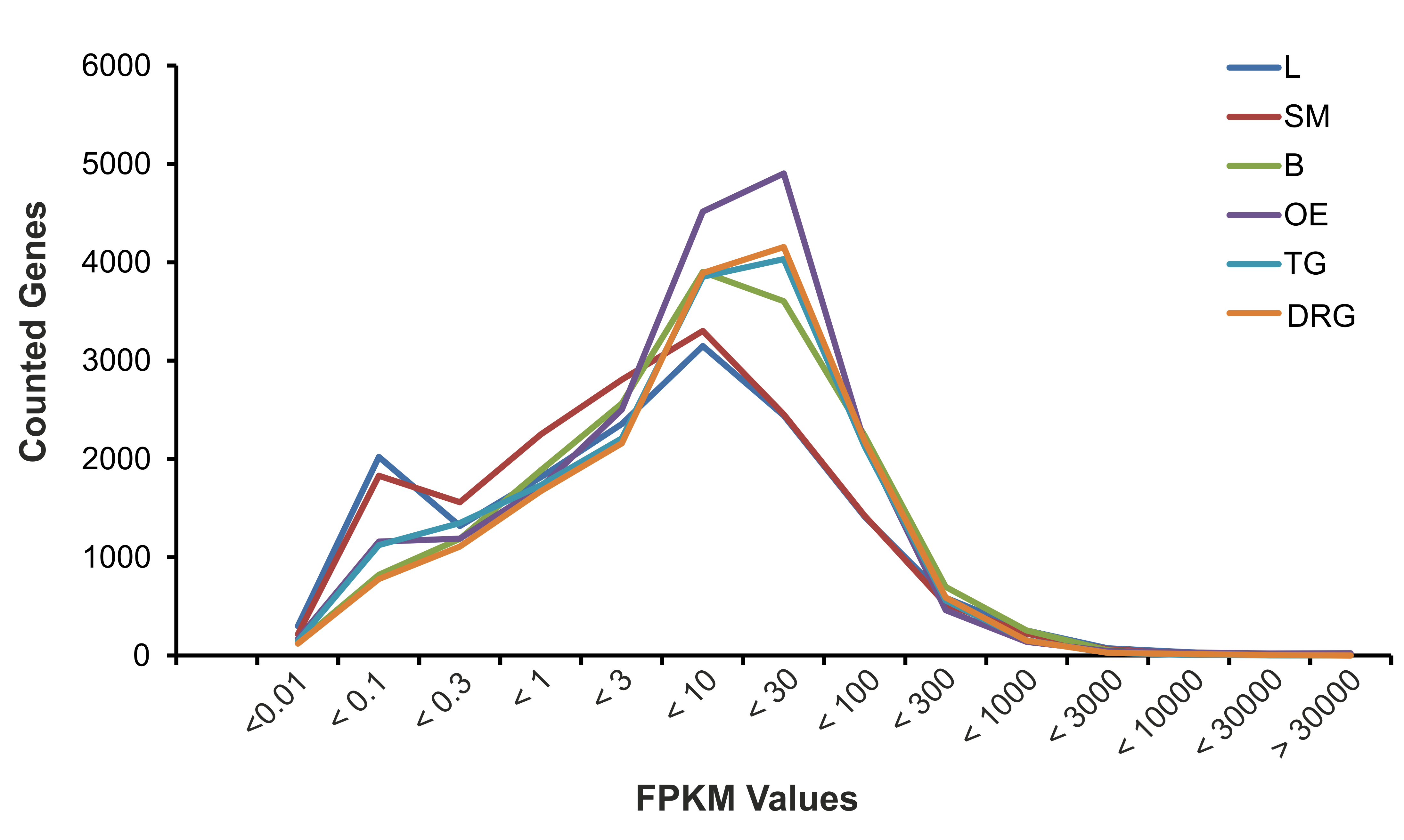

Supplement: Figure S2 — Distribution of FPKM values compared with the different tissues used. The highest numbers of genes are expressed between 1-10 FPKM in all tissues. There are fewer highly expressed genes with an FPKM of > 100. (TIF) [file pone.0079523.s002.tif]

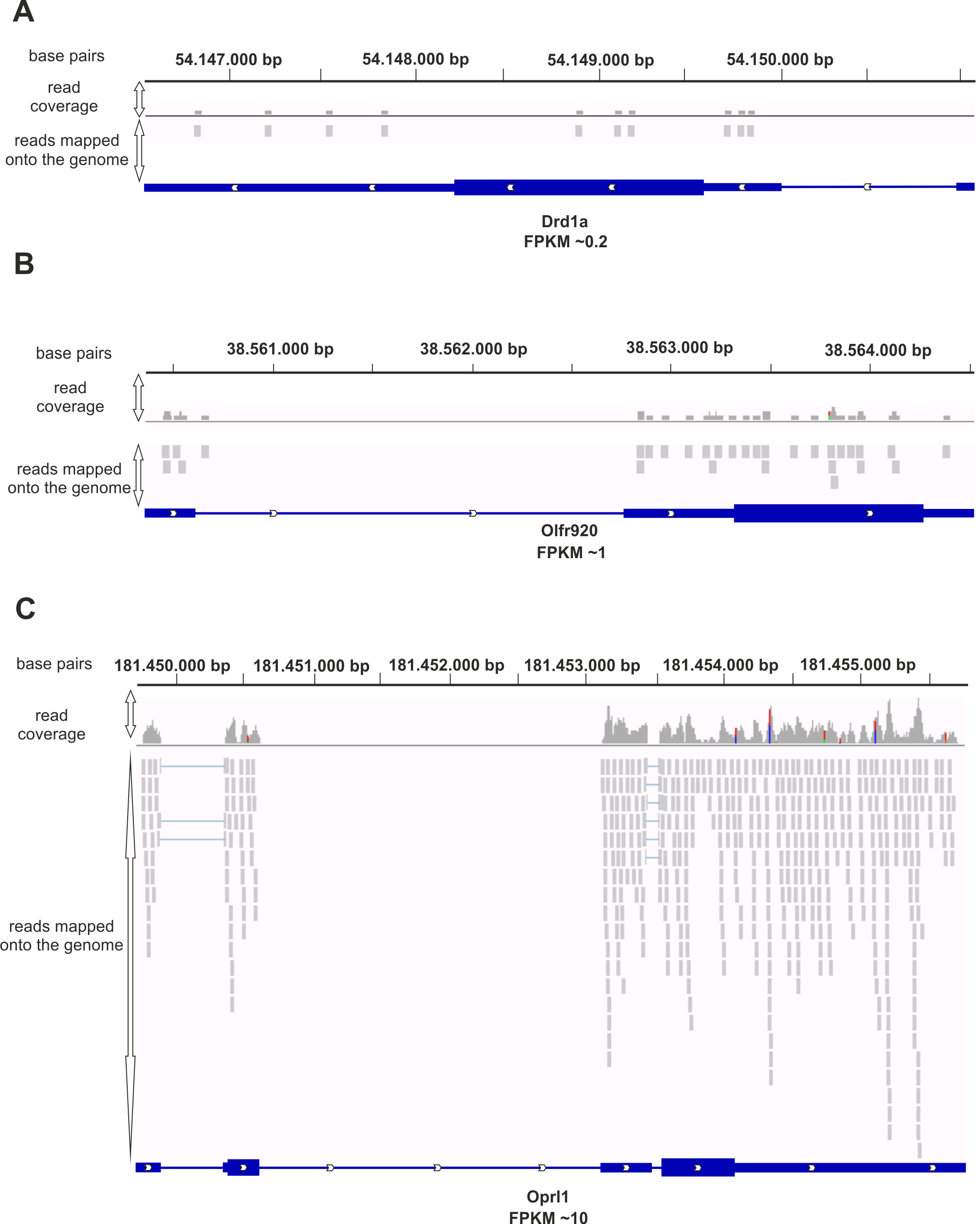

Supplement: Figure S3 — Integrative Genomic Viewer. Mapped reads for 1-kb large genes, which are expressed with 0.1 FPKM, 1 FPKM, and 10 FPKM. (TIF) [file pone.0079523.s003.tif]

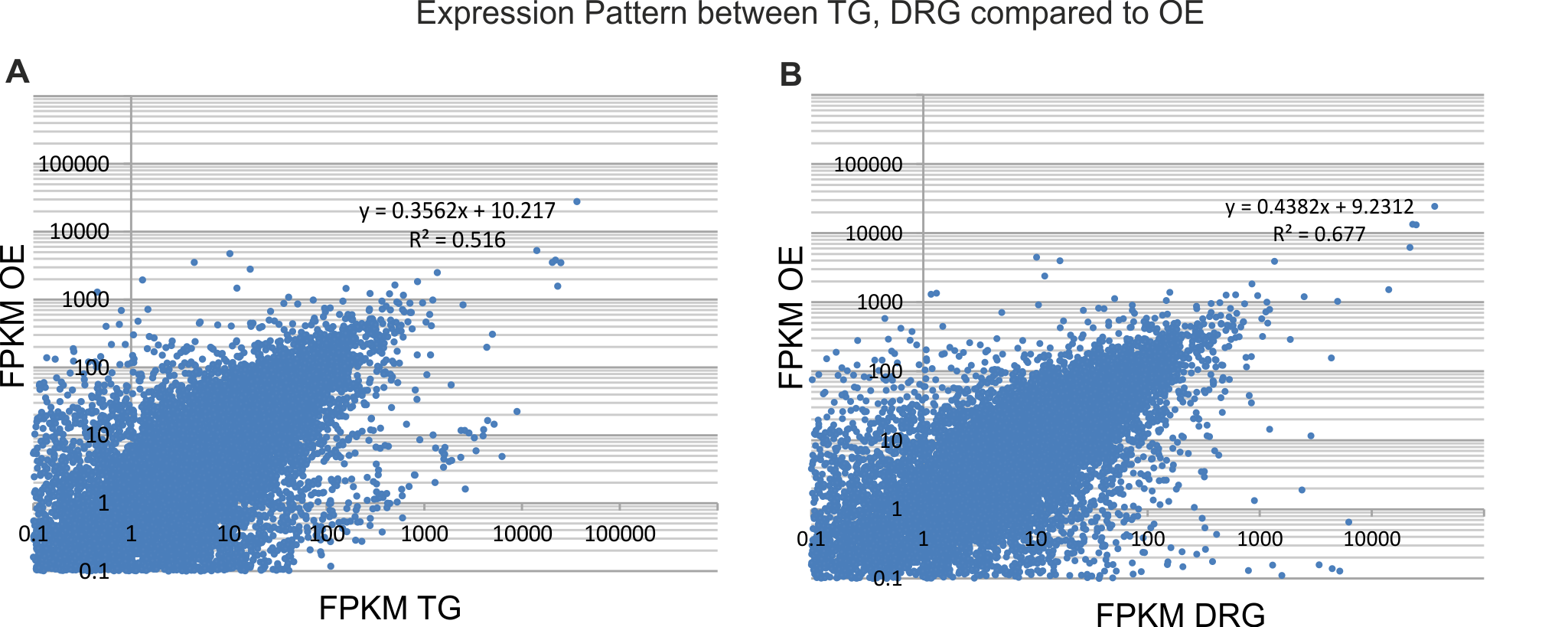

Supplement: Figure S13 — Comparison of the gene expression between the OE and TG and the OE and DRG. (TIF) [file pone.0079523.s013.tif]
